# Functional Yb-doped fiber with a bat-type refractive index distribution for beyond kilowatt all-fiber single-frequency laser amplification

**DOI:** 10.1038/s41377-025-01956-1

**Published:** 2025-08-12

**Authors:** Wei Li, Wei Liu, Yu Deng, Yisha Chen, Huan Yang, Qi Chen, Junjie Zheng, Hu Xiao, Zilun Chen, Zhiyong Pan, Pengfei Ma, Zefeng Wang, Lei Si, Shanhui Xu, Jinbao Chen

**Affiliations:** 1 College of Advanced Interdisciplinary Studies, National University of Defense Technology, Changsha, 410073 China; 2 Nanhu Laser Laboratory, National University of Defense Technology, Changsha, Hunan 410073 China; 3 Hunan Provincial Key Laboratory of High Energy Laser Technology, National University of Defense Technology, Changsha, Hunan 410073 China; 4 School of Physics and Optoelectronics, South China University of Technology, Guangzhou, 510640 China

**Keywords:** Fibre lasers, Nonlinear optics

## Abstract

High-power single-frequency fiber lasers with diffraction-limited spots are indispensable for a wide range of photonic applications and are particularly in advanced detection and sensing technologies. However, the simultaneous achievement of kilowatt-level output power and diffraction-limited beam quality has remained elusive in all reported single-frequency fiber laser systems to date, primarily due to limitations imposed by the stimulated Brillouin scattering (SBS) effect and transverse mode instability (TMI) effect. In this study, we demonstrate the design and manufacturing of an ultra-low numerical aperture (NA) functional Yb-doped fiber featuring a bat-type refractive index distribution, specifically engineered for single-frequency laser amplification. In the fabrication, we implemented multiple chelate gas filling and particle deposition iterations, leading to an active fiber with a bat-type refractive index distribution. The unique capabilities of this large mode area and high-order modes leakage fiber (HOMLF) were demonstrated by stably amplifying the single-frequency laser with more than one kilowatt output power and near single mode beam quality (M_x_^2^ = 1.10, M_x_^2^ = 1.18) for the first time. This fiber design advances the leap forward in single-frequency fiber lasers, which could contribute as a novel and efficient laser amplification technique for the next generation of gravitational wave detection systems.

## Abstract (web summary)

In this study, we demonstrate the design and manufacturing of an ultra-low numerical aperture functional Yb-doped fiber featuring a bat-type refractive index distribution, specifically engineered for realizing kilowatt single-frequency laser.

## Graphical abstract

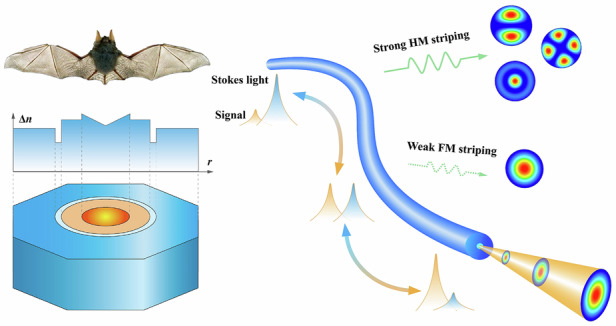

## Introduction

High-power single-frequency laser with diffraction-limited spot is the optimal light source for gravitational wave detection (GWD), remote communication, and other frontier scientific applications^1–7^. Fiber laser systems, renowned for their structural flexibility, ease of maintenance, and straightforward system cleanliness^8^, offer a promising pathway to achieving high-power single-frequency lasers. When coupled with advanced beam quality and noise control strategies, fiber laser systems are poised to deliver a reliable and high-performance light source for these demanding applications. The Laser Zentrum Hannover, which currently supplies the laser source for LIGO, has emphasized that kilowatt-class, ultra-low-noise single-frequency fiber lasers are pivotal for next-generation gravitational wave detection systems^9^. This sentiment was further underscored in a 2019 Nature in focus news article, which outlined a major upgrade plan for LIGO aimed at significantly enhancing its detection capabilities. One of the key elements in this plan is the power enhancement of single-frequency fiber lasers^10^. However, the power scaling of single-frequency fiber laser is roughly challenging, attributed to the inherent contradictions of the comprehensive suppression to the stimulated Brillouin scattering (SBS) and the transverse mode instability (TMI) effects within conventional step-index active fiber (SIF) assisted amplifiers^11,12^.

**Fig. 1:**
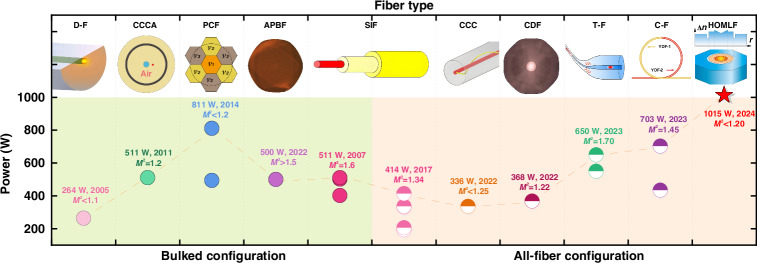
Research progress of all-fiber single-frequency lasers. (D-shaped fiber (DF), Chirally-Coupled-Core air-clad fiber (CCCA), photonic crystal fiber (PCF) and all-solid photonic bandgap fiber (APBF), step-index fiber (SIF), Chirally-Coupled-Core fiber (CCC), confined-doped fiber (CDF), tapered active fiber (T-F), cascade active fibers (C-F))

Specially designed fibers are an important milestone in the development of single-frequency fiber lasers, which has profoundly impacted the comprehensive suppression of the SBS and TMI effects. Benefiting from the special acoustic and mode structure design in the specially designed fibers, single-frequency laser ushered in vigorous development in the last dozen years. Figure 1 demonstrates the classical progress made in recent years, encompassing both specially designed fibers and conventional SIFs-based systems^13–16^. Within space-coupled configurations, a diverse array of specially designed active fibers has been designed and successfully applied in single-frequency amplifiers. Notable examples include D-shaped fiber (D-F)^17^, Chirally-Coupled-Core air-clad fiber (CCCA)^18^, photonic crystal fiber (PCF)^19–21^ and all-solid photonic bandgap fiber (APBF)^22^, and so on. Among these, the acoustic and gain-tailored PCF proposed by C. Robin et. al performs the best in the suppression of SBS and TMI effects. Through additional temperature control, the SBS threshold of this PCF could be enhanced 2.8 times, as a result, an 811 W single-frequency laser with a beam quality of M^2^ < 1.2 was achieved in 2014, which is the power record for single-frequency fiber laser^21^. Even after a decade of development, accompanied by many other innovative fiber designs, this power record has still remained unsurpassed. Within all-fiber configurations, a variety of specially designed fibers have also been introduced, such as Chirally-Coupled-Core fibers (CCC)^23^, confined-doped fibers (CDF)^24^, tapered active fibers (T-F)^25,26^, cascade active fibers (C-F)^27^. Benefiting from the superiority of specially designed fibers in nonlinear effects and TMI effect suppression, the output power of the all-fiber single-frequency fiber laser was increased from 194 W in 2009 to 703 W in 2023 (the power record for all-fiber configuration), averaging 100 W enhancement every three years^28^. Besides, while all-fiber systems are highly favored for their compactness, integrability, and reliability, power scaling beyond 500 W often comes at the cost of significant beam quality degradation and simultaneously requires ancillary strategies such as stress/temperature gradient control and polarization management, which introduce system complexity and hinder practical applications^15,24,27^.

In this paper, we presented a novel design of high-order modes leakage fiber (HOMLF) and evaluated its performance in single-frequency laser amplification. In contrast to previously reported single-frequency laser systems utilizing specially designed fibers, the proposed HOMLF enables a significant enhancement in output power, achieving kilowatt-level performance without the need for auxiliary strategies. Notably, the beam quality is maintained at M² < 1.2 even at an output power of 1015 W.

## Results

### Basic consideration

Conventional studies pointed out that various fiber parameters could simultaneously change the SBS and TMI thresholds of high-power single-frequency fiber amplifiers, such as the core/cladding diameter, ratio of doping area, cladding pump absorption coefficient, or effective fiber length. However, changes in these parameters had the opposite effect on the suppression of SBS and TMI effects^11,12,29^. Therefore, the comprehensive suppression of SBS and TMI effects necessitates analysis of the physical properties of fiber optic waveguides to find a new breakthrough. In the double-clad fibers, the effective mode area of the fundamental mode and the bending loss coefficient of *LP*_*11*_ mode could be simply described as^30^:

1 $$V=\frac{2\pi aNA}{\lambda }$$

2 $${M}_{eff}\approx \pi {a}^{2}{\left(0.65+\frac{1.619}{{V}^{3/2}}+\frac{2.879}{{V}^{6}}\right)}^{2}$$

3 $${\alpha }_{coil}=\frac{{K}_{m}\exp [-2{W}^{3}R/(3{a}^{3}{\beta }^{2})]}{{W}^{3/2}\sqrt{aR}{V}^{2}}$$

Where *V* is the normalized cutoff frequency, *NA* is the numerical aperture, *M*_*eff*_ is the effective mode area, *α*_*coil*_ is the bending loss coefficient, and *K*_*m*,_ *W, β* are the characteristic parameters related to *LP*_*mn*_ mode. *a* and *R* response to the fiber core and bending diameter. Equations (1–3) show that, with the decrease of *NA*, the effective mode area and bending loss will increase which are potential to suppress the SBS and TMI effects at the same time (detailed simulation results are shared in the supplementary section I).

**Fig. 2:**
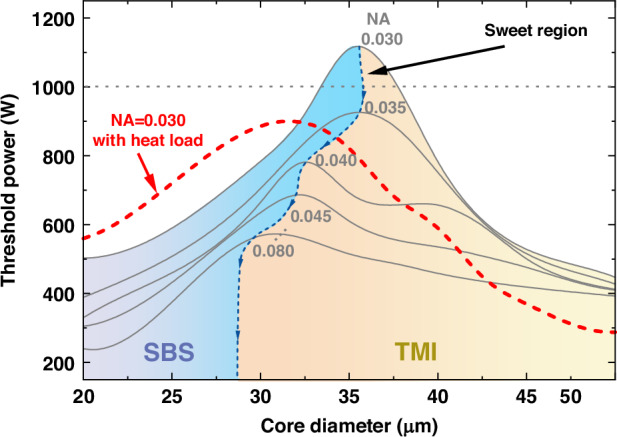
The simulated threshold power of the single-frequency amplifier

Then, based on the SBS and TMI theoretical evaluation models we shared in the supplementary section II, the maximum output power of the single-frequency fiber amplifiers with different *NAs* and fiber core sizes is calculated. The key parameters setting in the theoretical evaluation models are demonstrated in Table 1 (where *α*, *L*_*a*_, *L*_*p*_, *d*_*core*_, *NA*, *P*_*s*_, *λ*_*s*_, *λ*_*p*_ demonstrate the absorption coefficient, the length of the active fiber, the length of the passive fiber, the core diameter, the numerical aperture, the injected seed power, the wavelength of the seed laser and the pump laser). The calculated results are shown as the gray lines in Fig. 2, which demonstrates that, with the enhancement of core diameter, the limitation of the output power will gradually change from the SBS effect to the TMI effect. Meanwhile, benefiting from the enhancement of the effective mode area of the fundamental mode and bending-loss coefficient of the *LP*_11_ mode brought by the decreasing of the *NA*, the threshold power of the single-frequency amplifier increases obviously. Accordingly, setting the *NA* and core diameter around 0.030 and 35 μm is a promissing design for achieving kilowatt single-frequency lasers. In our analytical discussion, we have set 0.03 as the minimum core *NA* threshold, considering that optical fiber designs with *NA* below this threshold may experience decreased longitudinal *NA* uniformity and unstable fiber performance during actual manufacturing processes^31^.

**Table 1:** The key parameters

Parameter	Value	Parameter	Value
*α*	7 dB/m	*L*_*a*_,	1.5 m
*L*_*p*_	0.1 m	*d*_*core*_	20-50 μm
*NA*	0.030-0.080	*P*_*s*_	10 W
*λ*_*s*_	1030 nm	*λ*_*p*_	976 nm

### Challenge induced by thermal load

However, in conventional SIFs associated high-power single-frequency fiber laser systems, the high thermal load per unit length resulting from high Yb doping concentrations and short fiber designs will lead to an increase in the core numerical aperture (NA), causing high-order modes (HOMs) to relocate into the core region. This relocation diminishes the advantage of the high HOMs loss coefficient introduced by the low *NA*, ultimately preventing the fiber from maintaining single-mode operation characteristics under high-power load conditions^32^. In the Supplementary Section II, we elaborate on the methodology for evaluating the thermal load in optical fibers by incorporating quantum defect and heat conduction equations and the specifical thermal load distribution is shared in the Supplementary Section IV. Theoretical simulation results indicate that thermal effects induce slight variations in the Brillouin gain coefficient and fundamental mode field area of the fiber, accompanied by a significant reduction in the high-order mode loss coefficient which results in the decrease of TMI threshold and ultimately lead to a decrease in the threshold power of the system. Specifically, we have given out the threshold evolution curve of a SIF with a NA of 0.03 when thermal load is considered as shared in Fig. 2 red dotted line. Analysis reveals that with consideration of the thermal load, the maximum threshold power of the simulated system is 19.4% lower than the ideal maximum threshold without thermal load. Therefore, simultaneously elevating the SBS and TMI thresholds above the kilowatt level remains challenge in addressing the degradation of high-order mode bending loss coefficient under high thermal load conditions.

### Fiber design inspiration

**Fig. 3:**
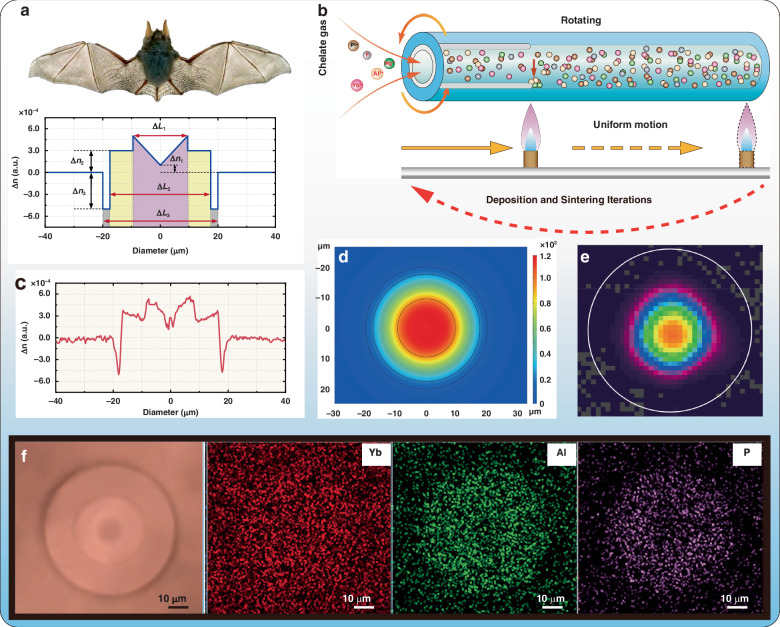
The design of the high-order modes leakage fiber. **a** The designed bat-type refractive index distribution (*ΔL*_*1*_ is the diameter within the center region, *ΔL*_*2*_ is the diameter within the stepped region, *ΔL*_*3*_ is the diameter within the gully region, *Δn*_*1*_ represents the refractive index difference between the center point of the center region and the cladding, *Δn*_*2*_ represents the refractive index difference between the stepped region and the cladding, *Δn*_*3*_ represents the refractive index difference between the gully region and the cladding). **b** The fabrication process. **c** The measured actual refractive index distribution. **d** Beam profile obtained from the finite element simulation. **e** The actual beam profile obtained from the beam quality monitor. **f** The electron micrograph image of the HOMLF fiber

Based on our group’s extensive theoretical and experimental investigations over the past decade on trench fibers^33^, confined-doped fibers^24^, and all-solid photonic bandgap fibers^34^, as well as in-depth studies on refractive index profiles^35^, we recognized that precise design of refractive index distribution holds promise for addressing the challenges imposed by thermal loads. Specifically, to maintain high HOMs loss characteristics under high thermal load conditions, we propose a bat-type refractive index profile design, as illustrated in Fig. 3a. The core area of this fiber design is subdivided into three regions: the center region (colored in purple, Yb-doped), the stepped region (colored in yellow, Yb-doped), and the gully region (colored in gray, non-Yb-doped) from the inner to the outer layer. According to the above simulation, the Yb-doped diameter (*ΔL*_2_) of this new type of fiber is set to be 35 μm. Other parameters, including *ΔL*_1_, *ΔL*_3_, *Δn*_1_, *Δn*_2_, and *Δn*_3_, are designed and selected while the following three performance optimizations are comprehensively considered: (i) Ensure that the equivalent *NA* of the designed fiber is kept around 0.030 to balance the SBS and TMI effects. (ii) More than 50% energy of the fundamental mode is concentrated in the center region to reduce the energy coupling coefficients of the fundamental mode and the *LP*_11_ mode, and avoid the distortion of fundamental mode caused by fiber bending^36,37^. (iii) uneagerly the *LP*_11_ mode is distributed close to the edge of the core to increase the bending loss of the *LP*_11_ mode while further attenuating the energy coupling between the *LP*_11_ mode and the fundamental mode. The detailed analysis is shared in the supplementary section III, which indicates that when the mode field area of the *LP*_01_ mode is greater than 651.0 μm^2^, the bending loss coefficient of the *LP*_11_ mode is higher than 27.5 dB/m, and more than 50% energy of the fundamental mode is concentrated in the center region of the fiber core, the fiber can theoretically achieve a high-beam-quality kilowatt single-frequency laser with consideration of thermal load.

The finite element simulation illustrates that setting the pivotal parameters as Table 2 shows, will be a feasible choice to achieve high-power single-frequency lasers. For this fiber design, the effective mode field area of the fundamental mode is calculated to be 669.0 μm^2^ and the bending loss coefficient of the *LP*_11_ mode is calculated to be 47.5 dB/m according to the simulation. Figure 3d is the output beam profile obtained from the finite element simulation. The central power share ratio is calculated to be 53.4%, and 82.2% power share of the *LP*_11_ mode is distributed outside the center region. The advantages of this HOMLF over conventional low-NA SIFs in mitigating performance degradation caused by thermal loads are discussed in detail in Supplementary section IV.

**Table 2:** Parameters value

Parameter	Value	Parameter	Value
Δ*L*_1_	19 μm	Δ*n*_1_	0.0001
Δ*L*_2_	35 μm	Δ*n*_2_	0.0003
Δ*L*_3_	40 μm	Δ*n*_3_	−0.0005

### Implementation

The HOMLF is subsequently fabricated using the modified chemical vapor deposition (MCVD) in conjunction with the chelate gas deposition technique as demonstrated in Fig. 3b. After several attempts to continuously optimize the ratio of dopant ions to enhance the performance of the HOMLF in single-frequency laser amplification, the refractive index distribution of the fiber finally obtained is shown in Fig. 3c. The center/stepped/gully-cladding diameter of this high-order modes leakage fiber is measured to be 18.5/34.6/40.9/250.2 µm. The relative refractive index of the high fluoride ion doped gully region was measured to be -0.0005 while it was 0.0003 for the stepped region. As for the center region, the relative refractive index is fabricated into a concave with the maximum/minimum relative refractive index of 0.0005/0.0001. Importing the measured refractive index distribution into the finite element simulation model, the effective mode field area of the fundamental mode is calculated to be 670.9 μm^2^, and the energy share of the central fundamental mode is 51.7%. Additionally, the bending loss coefficient of the *LP*_01_ mode is calculated to be 0.011 dB/m while it’s 36.4 dB/m for the *LP*_11_ mode. Besides, the normalized cutoff frequency (*V)* is calculated to be 3.63, So only *LP*_01_ mode and *LP*_11_ mode are supported in this fiber. Moreover, based on the practical refractive index profile, we analyzed the thermal load distribution and the bending loss distribution of the *LP*_11_ mode along the fiber, as well as the power evolution of the *LP*_01_ and *LP*_11_ modes. Detailed results can be found in Section IV of the supplementary.

Further, we tested the actual ion distribution and ion content percentage of the fabricated high-order modes leakage fiber by using an electron probe microanalyzer. The two-dimensional energy-dispersive X-ray (EDX) mapping distribution of Yb, Al, and P ions is illustrated in Fig. 3f. According to the EDX mapping distribution, the molar shares of these three major doping ions in the fiber core are measured to be 0.16%, 1.40%, and 1.09%, respectively. Subsequently, we tested the high-order modes leakage fiber for light transmission in a fiber length of 30 m. With a single-mode laser inserted in, the high-order modes leakage fiber enabled to output laser with stable fundamental mode. As shown in Fig. 3e, the profile at the focused spot kept a good Gaussian morphology and the beam quality (M^2^ value) of the output laser was measured to be ~1.15, indicating that this fiber has a priority in fundamental mode transmission, even though it possesses an effective mode field area as large as 670.9 μm^2^.

### System construction

**Fig. 4:**
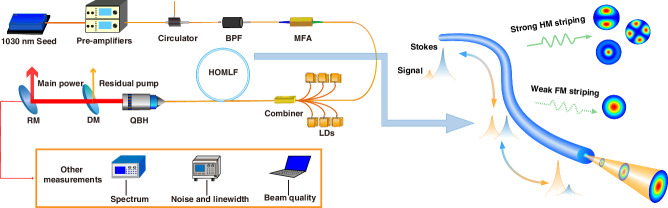
The configuration of the single-frequency fiber laser system. (MFA: band-pass filter; MFA: mode-filed-adaptor; LDs: laser diodes; HOMLF: high-order mode leakage fiber; QBH: quartz block holder ; DM: dichroic mirror; RM: reflecting mirror)

To explore the capacity of this highly-Yb-doped high-order modes leakage fiber in generating higher-power single-frequency lasers, a classical single-frequency master oscillator power amplifier is conducted to examine its performances, as shared in Fig. 4. The single-frequency seed is a distributed feedback Bragg grating laser with a central wavelength and linewidth of 1029.5 nm and 900 Hz. This seed is first amplified to ∼10 W by two commercial cascaded pre-amplifiers. After the Pre-amplifiers, a high-power circulator is inserted to export the backward power for the SBS power record. Near behind, a band-pass filter (BPF) with a bandwidth of 1030 ± 4 nm is employed to remove the spectral sideband noise, which will facilitate the suppression of amplified spontaneous emission (ASE) in the main amplifier. A mode-filed-adaptor (MFA, 10/125 µm to 20/250 µm) is then fused, to inject the amplified signal laser into the main amplifier.

The main amplifier is constructed in a forward pumping scheme and six 370 W 976 nm laser diodes (LDs, the core/cladding diameter of the pump deliver fiber is 135/155 μm) are employed to provide pump power via a (6 + 1) × 1 pump/signal combiner. In this combiner, the core/ cladding diameter of the signal input and output port is 30/250 µm. A 1.5 m high-order modes leakage fiber is fused at the output port of the combiner to provide pump power for the single-frequency laser system. The absorption coefficient of this active fiber is measured to be 7 dB/m@ 976 nm. The fiber is coiled on a water-cooling plate with a bending radius of 0.265 m. Finally, the amplified laser is output through a quartz block holder (QBH), in which a cladding light stripper and an endcap are integrated. Here, the total length of the passive fiber between the active fiber and the endcap is controlled as short as 8 cm for better suppression of the SBS effect. The output laser is then collimated and transmitted to a dichroic mirror (DM) to remove the residual pump. A reflecting mirror (RM) with a reflectivity of 99.7% is then placed in the main light path, and the major signal power is reflected to a power meter for power recording while the transmitted 0.3% signal laser is transferred to the high-precision measuring devices for other characteristic parameters recording, such as spectrum, linewidth, intensity noise as well as beam quality.

### Measurement of key performance parameters

**Fig. 5:**
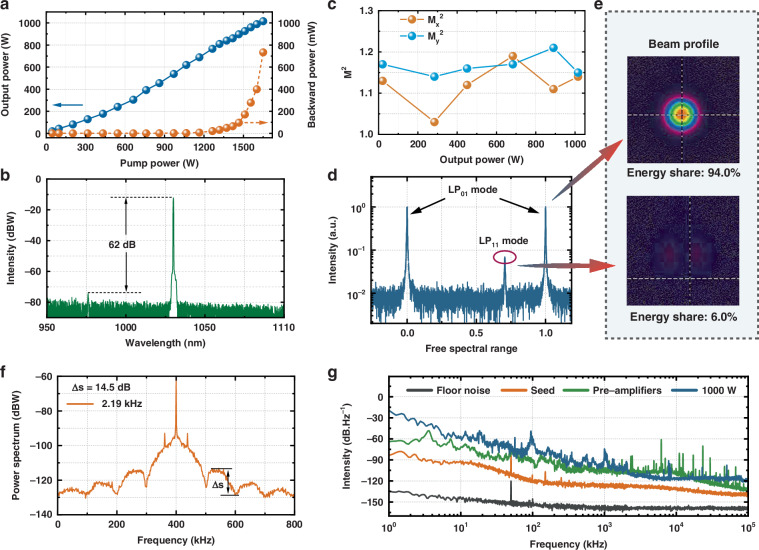
Main parameters of the output laser. **a** The output power and backward power versus the pump power. **b** Output spectrum. **c** Beam qualities (M^2^ value). **d** The mode scan result. **e** The beam profile recorded after the optical cavity. **f** Self-heterodyne spectrum; **g** The RIN within 100 kHz

Based on the above-mentioned amplifier, the superiorities of the high-order modes leakage fiber in single-frequency laser amplification are testified. Benefitting from the large mode filed area and high-order modes loss feature, the SBS and TMI effects are effectively suppressed in the power amplification process. When the injected pump power increases to 1650 W, the output power of the single-frequency laser reaches 1015 W. The power ramping-up process is displayed in Fig. 5a, in which it could be found that the optic-to-optic conversion ratio of the system performs in a slightly increasing trend due to the pump laser wavelength drifting. Once the pump power is improved beyond 800 W, the pump wavelength will be locked at 976 nm, and the optic-to-optic conversion ratio will stabilize at 61.1%. As for the backward power, which is usually used as a criterion to reveal the intensity of the SBS effect, it starts with a linear increase but begins to nonlinearly grow when the output power is amplified beyond 900 W, indicating the occurrence of the SBS effect. At the maximum output power (1015 W), the backward power is measured to be 746 mW. The spectrum property of the output laser is also recorded at 1015 W, as demonstrated in Fig. 5b. Although the total absorption coefficient of the amplification system is only about 10 dB, the pump laser in the output laser is well-stripped attributed to the cooperative work of the cladding light stripper and dichroic mirror. As a result, the intensity of the signal laser is 62 dB higher than the pump laser while no ASE component is observed in the spectrum.

In addition, the beam qualities of the output signal laser are measured from 30 W to 1015 W, and the measured results are demonstrated in Fig. 5c. Accordingly, the M^2^ value is consistent below 1.2 at all power levels, confirming that the high-order modes leakage fiber possesses superior single-mode operation ability even at high power operation. To provide a more precise characterization of the beam quality, we adopt the optical cavity-based mode scanning method to accurately measure the mode content of the output laser^38^. The mode scan spectrum and the scanning beam profile of the optical cavity recorded at kilowatt level are shared in Fig. 5d, e. According to the mode scan spectrum and the recorded beam profile, the output laser only consists of the *LP*_01_ and the *LP*_11_ mode, with the *LP*_01_ mode accounting for 94.0% of the total energy.

Furthermore, the linewidth and noise characters of the output signal laser are measured. Figure 5f is the self-heterodyne spectrum of the output laser at 1015 W. According to the wave height of the second harmonic of the self-heterodyne spectrum, the linewidth of the signal laser is calculated to be 2.19 kHz. Compared with the seed laser, the linewidth of the signal laser widened more than two times in the amplification process, which was attributed to the pump-introduced noise and the SBS effect. The evolution of linewidth is provided in Supplementary Section V. Secondly, the relative intensity noise (RIN) of the seed laser, cascaded pre-amplifiers, and output laser is measured, as plotted in Fig. 5g. It could be seen that the seed laser exhibits an intensity noise of approximately -140 dB at the frequency of 100 kHz. However, after passing through the pre-amplifier and main amplifier, the output laser shows significant noise degradation in the 1–100 kHz band, due to the pump noise, the thermal noise, and the ambient noise introduced by the power amplification system. Finally, the intensity noise of the laser at 100 kHz degrades to approximately -120 dB, at an output power of 1000 W. Further applying proven noise suppression strategies such as hybrid proportional-integral-derivative (PID) feedback loop, high-power photodiode array, and squeezed vacuum states et al. is expected to achieve enhanced reductions in the intensity noise of the output laser, and advance the frontier scientific applications of single-frequency fiber lasers^39–42^.

In addition, the amplifier demonstrates exceptional long-term stability, with a comprehensive evaluation of key output laser parameters provided in Supplementary VI. Key performance metrics include reliable operation at the kilowatt-level output, demonstrating a power fluctuation of approximately 2.2%. During long-term high-power operation, the amplifier maintains a good polarization state, evidenced by a polarization extinction ratio of 19 ± 1.5 dB. The pointing noise of the output laser is quantified at ~1.8 × 10^-5^ Hz^1/2^ at 10 kHz in both the *x* and *y* directions. And the phase noise characteristics of the seed laser within the 1–10 kHz range are consistently retained throughout the power amplification process. These results provide valuable insights for the optimization of the light sources intended for cutting-edge applications such as gravitational wave detection.

## Discussion

The work takes a concrete step in the power scaling of single-frequency fiber lasers by developing a specially designed refractive index distribution to overcome the tradeoff of the comprehensive suppression of the SBS effect and TMI effect, a prevalent and consistent challenge in high-power single-frequency fiber amplifiers. It proposes and experimentally implements an active fiber with bat-type refractive index distribution to achieve the high HOMs bending loss feature and large effective mode field area character; it also experimentally demonstrates that employing active fibers with a bat-type refractive index distribution and ultralow *NA* constitutes an effective approach for achieving a significant breakthrough in single-frequency fiber laser performance. Furthermore, our fiber hierarchical design paradigm, progressing from basic physics analysis to identification of critical challenges, theoretical simulation-driven tolerance assessment, and experimental validation, establishes a systematic framework that offers critical guidance for advancing high-power laser system development incorporating specially designed fibers, particularly for systems constrained by nonlinear optical effects and thermo-optic limitations.

Through further optimization of the transverse refractive index distribution and gain field distribution of the fiber incorporation with the longitudinal mode field radius distribution, new advancements in power scaling for single-frequency laser systems can be anticipated. And for comprehensive multi-parameter optimization of fiber design, integrating artificial intelligence (AI) algorithms into the fiber design process represents a transformative opportunity. AI-driven optimization enables multi-parameter fiber design, facilitating rapid prototyping and performance improvements. Leveraging machine learning to analyze and predict fiber behavior under various conditions is expecting to accelerate the development of next-generation fibers tailored to specific applications, and propel fiber lasers to more pioneering scientific research areas.

The bat-type refractive index distribution design also exhibits excellent compatibility with existing fiber structures, ensures seamless integration into current laser systems, paving the way for cost-effective mass production. Additionally, this refractive index distribution offers a novel approach to comprehensively suppressing nonlinear effects and transverse mode instability (TMI), making it highly promising for broader applications in fields such as broadband laser amplification and ultrafast laser systems.

## Materials and methods

### Details in fiber fabrication process

Firstly, multiple high-purity raw particles are prepared, including SiCl_4_, POCl_3_, Yb (Thd)_3_, AlCl_3_, SiF_4_, et al. Then, the MCVD process starts with a standard F-300 Heraeus fused silica tube, which is used for the deposition of the uniform chelate gas mixture consisting of silica and other doped ions. This silica tube is mounted on two synchronized rotating chucks in a bedroom glass machine. One end of the silica tube is connected to the chemical feedstock supply system for mixing and feeding raw particles into the silica tube at a controlled flow rate, and the other end of the silica tube is connected to the reaction off-gas and dust treatment equipment. Afterward, the prepared chelate gas which contains elements such as Yb/Al/P ions are flushed into the silica.

The oxygen-hydro flame torch is arranged under the silica tube and controlled to move along the direction of gas flow in the tube. With the high-temperature environment brought by the oxygen-hydrogen flame torch, chemical reagents such as silicon tetrachloride inside the tube will be oxidized and/or hydrolyzed to produce submicron-sized silica glass particles containing the dopant ions, which will be deposited on the inner surface of the silica tube downstream of the hot zone. At the same time, the silicon tube is controlled to rotate at a rate of several tens of revolutions per minute for uniform distribution of the deposited ions throughout the inner wall of the silica tube. In order to have precise control over the refractive index distribution of the fiber core, the ionic ratios of the gas mixture, the flow rate, and the moving speed of the oxygen-hydro flame torch are precisely formulated. For each time the oxygen-hydro flame torch translation passes, the multielement gas would be sintering into a ~ 1μm-thick transparent glass film with an elaborate refractive index. Further applying multiple iterations of deposition and sintering, a core refractive distribution with a gully region, stepped region, and smooth center concave region is obtained. After completing all iterations of deposition and sintering, the silica tube underwent consolidation and collapsed into a transparent solid performed at a higher temperature. Finally, the preform was cased to expand the cladding radius, and the periphery of the expanded perform was machined into an octagonal shape with a surface polished and then stretched to a fiber in the special fiber drawing tower.

### Major parameters measurement methods

The beam quality of the output laser is measured using a camera-based beam profile analysis system. The D4*σ* method is employed to monitor the variation trend of the laser spot size near the focal point. By fitting this spot size variation curve, the laser divergence angle is determined, thereby enabling the calculation of the M² factor according to the ISO 9001 standard.

The mode composition of the output laser is characterized by employing an optical cavity. Firstly, the beam is polarization-split, with the S-polarized component coupled into a piezoelectric-actuated tunable optical cavity. The cavity length modulation via a triangular-wave excitation enables resonant mode discrimination, from which both mode power signatures and corresponding transverse beam patterns are extracted at the output port.

The linewidth measurement is based on the self-heterodyne method. Firstly, the weak signal laser behind the reflecting mirror is coupled into a linewidth analyzer through a single-mode fiber. The linewidth analyzer consists of a Mach–Zehnder interferometer (The length of the single-mode delayed fiber is 2 km), a photodetector (Thorlabs PDA05CF2), and a signal analyzer (PXA Signal Analyzer N9030A). Then according to the recorded self-heterodyne beat spectrum, the linewidth of the output laser can be obtained. The detailed theory of linewidth analysis is provided in Supplementary Section V.

The relative intensity noise of the output laser is measured by a photodetector-based noise analysis system. Firstly, the weak signal laser behind the reflecting mirror is coupled into a single mode fiber and directed to the photodetector (Thorlabs PDA05CF2) for optoelectronic conversion. Then the conditioned electrical signal was AC-coupled via a capacitor and subsequently fed into a signal analyzer ((Dynamic Signal Analyzer-785), where the intensity noise spectrum of the laser output is recorded.

## Supplementary information

Supplemental File

## Data Availability

The data underlying the results presented in this paper are not publicly available at this time but may be obtained from the authors upon reasonable request.
