# Supplementary material for: Functional Yb-doped fiber with a bat-type refractive index distribution for beyond kilowatt all-fiber single-frequency laser amplification
Source: Light Sci Appl. 2025 Aug 12;14:271. doi: 10.1038/s41377-025-01956-1 (PMC12343874; doi:10.1038/s41377-025-01956-1)
Supplement: Supplementary file 1 — Supplemental File [file 41377_2025_1956_MOESM1_ESM.docx]

Supplementary Information for

Functional Yb-doped fiber with a bat-type refractive index distribution for beyond kilowatt all-fiber single-frequency laser amplification

Wei Li^1^, Wei Liu^1,2,3^, Yu Deng^1^, Yisha Chen^1,2^, Huan Yang^1,2^, Qi Chen^1^, Junjie Zheng^4^, Hu Xiao^1,2,3^, Zilun Chen^1,2,3^, Zhiyong Pan^1,2,3^, Pengfei Ma^1,2,3,*^, Zefeng Wang^1,2,3^, Lei Si^1,2,3^, Shanhui Xu^4,5^ and Jinbao Chen^1,2,3^

^1^College of Advanced Interdisciplinary Studies, National University of Defense Technology, Changsha 410073, China

^2^Nanhu Laser Laboratory, National University of Defense Technology, Changsha, Hunan, 410073, China

^3^Hunan Provincial Key Laboratory of High Energy Laser Technology, National University of Defense Technology, Changsha, Hunan, 410073, China

^4^ School of Physics and Optoelectronics, South China University of Technology, Guangzhou 510640, China

^5^ [flxshy@scut.edu.cn](mailto:flxshy@scut.edu.cn)

These authors contributed equally: Wei Li, Wei Liu

*Corresponding author: shandapengfei@126.com

**Table of Contents:**

Section I: The calculated results of the effective mode area and *LP*_11_ mode bending loss coefficient by applying the classical estimate equations.

Section II: Threshold simulation models for SBS and TMI effects analysis

Section III: Fiber parameters design and tolerance analysis

Section IV: Analysis of bending loss and power evolution under strong thermal load

Section V: The method for linewidth estimation and linewidth measure result

Section VI: Characteristic parameters of the output laser

**Section I**

In high-power single-frequency fiber lasers, the improvement of the output power and beam quality is mainly limited by the stimulated Brillouin scattering (SBS) and/or the transverse mode instability (TMI) effects. Previous studies have pointed out that enlarging the effective mode area of the fundamental mode can reduce the intensity of the laser per unit area and lead to a higher SBS threshold power^1^. Besides, previous studies have also pointed out that a larger bending loss coefficient would be helpful to suppress the energy coupling between the fundamental mode and higher-order modes and lead to a higher TMI threshold^2^. In this section, we did a detailed calculation on the changes in the effective mode field and the bending loss coefficient of *LP*_11_ mode caused by numerical aperture alterations by applying the classical effective mode field and *LP*_11_ mode bending loss coefficient estimating equations. The result demonstrates that decreasing the core *NA* is a promising method to simultaneously enhance the SBS and TMI threshold power.

Figure S1 demonstrates the simulated results when the core diameter is set at 20 μm, 30 μm, 40 μm, and 50 μm. Here, we only investigate the bending loss coefficient of *LP*_11_ mode, because *LP*_11_ mode is the dominant mode of coupling to the fundamental mode when the TMI effect occurs. Besides, the bending loss coefficient of *LP*_11_ mode given out in figure S1(b) is calculated when the bending loss coefficient of *LP*_01_ mode is fixed at -0.1 dB·m^-1^. Figure S1(a-b) demonstrates that the effective mode area of the *LP*_01_ mode could be enlarged for times while the *LP*_11_ mode bending loss coefficient could be exponentially enhanced when the core *NA* decreases from 0.08 to 0.02. Therefore, decreasing the core *NA* of active fiber will be a promising method to suppress the SBS and TMI effects at the same time. In addition, it is important to note that, when the core *NA* is controlled below 0.03, there is a high risk that the core *NA* will be unstable along the fiber in the practical fabrication process.

| 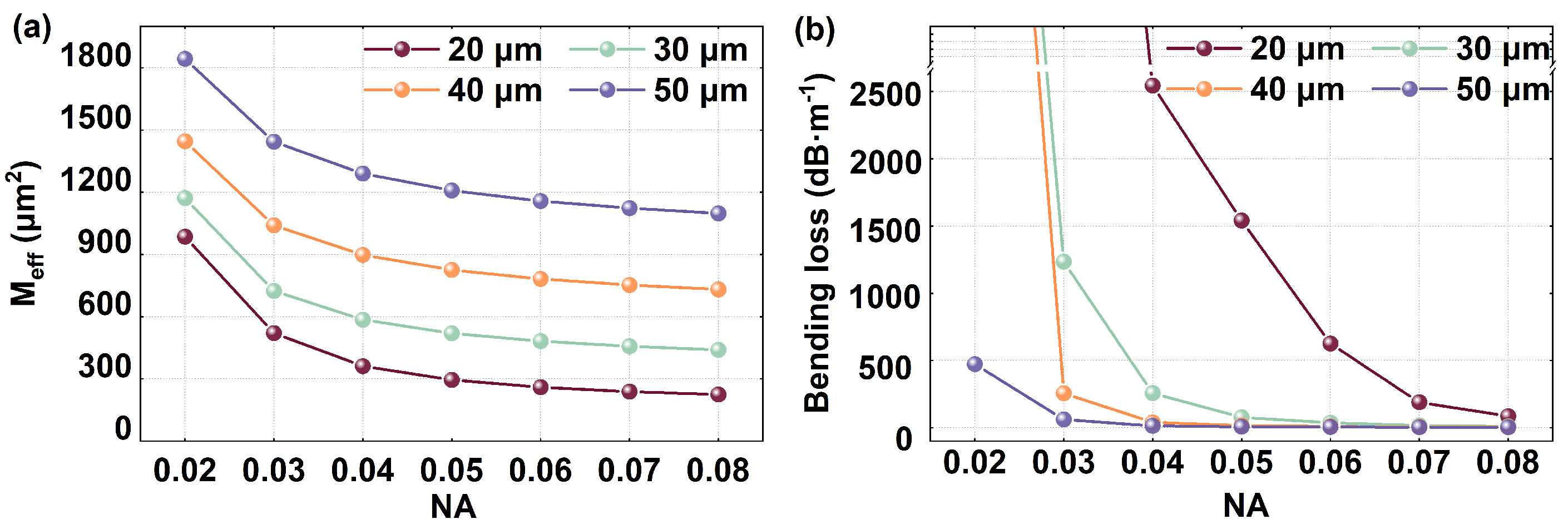 |  |
| --- | --- |

**Fig. S1** Change trend of (a) the effective mode field of *LP*_01_ mode and (b) the bending loss coefficient of the *LP*_11_ mode with the numerical apertures.

**Section II**

The theoretical analysis of SBS and TMI threshold power is an important guide for the design of active fibers. In this section, we gave out our simulation models for SBS and TMI threshold calculations. Based on these two models, the change of the threshold power of the single-frequency fiber lasers could be obtained as shown in the manuscript, figure 2.

Following are the theoretical models for the stimulated Brillouin scattering (SBS) and the transverse mode instability (TMI) thresholds power calculation, which we applied in the paper.

1. *Theoretical models for SBS threshold power calculation*

In order to describe the evolution of the SBS effect in optical fibers, nonlinear interaction between the signal light, the Stokes light, and the acoustic wave should be considered. The differential equations in time (*t*) and position (*z*) that relate these waves can be written as ^3^:

$$\left\{\begin{matrix} \frac{\partial E_{s}}{\partial z}+v_{s}\frac{\partial E_{s}}{\partial t}\text{=}\frac{1}{2}(g_{s}-\alpha_{s})E_{s}\text{+}i\kappa E_{B}Q \\ \frac{\partial E_{B}}{\partial z}+v_{B}\frac{\partial E_{B}}{\partial t}\text{=}\frac{1}{2}(g_{B}-\alpha_{B})E_{B}\text{+}i\kappa E_{S}Q \\ \frac{\partial Q}{\partial z}+v_{A}\frac{\partial Q}{\partial t}\text{=}-\frac{1}{2}\Gamma_{B}Q\text{+}i\frac{\kappa}{A_{ao}}E_{S}E_{B}^{*}+f \end{matrix}\right.$$

where *E_s_*、*E_B_* and *Q* represent the complex amplitude of the signal, Stokes light, and the acoustic field. *v_s_* and *v_B_* are the group velocities of the signal and Stokes light, respectively, while *v_A_* is the speed of sound，*γ_e_* is the electrostriction constant of quartz, and *ρ*_0_ is the density of the quartz. *A_ao_* is the overlapping area of the acoustic and optical fields, and since the fundamental mode area of the acoustic wave field is much larger than that of the optical wave field, *A_ao_* can be approximated replaced by the effective mode area of the signaling light. *g_s_* and *g_B_* are the active gains of the signal and Brillouin-Stokes light, which satisfy the following expressions:

$$g_{i}=(\sigma_{ai}+\sigma_{ei})N_{2}-\sigma_{ai}N_{0}\text{, } i=s,B$$

$$\pm\frac{\partial P_{p}^{\pm}}{\partial z}+\frac{1}{v_{p}}\frac{\partial P_{p}^{\pm}}{\partial t}=-\alpha_{p}P_{p}^{\pm}-\Gamma_{p}\left\{\sigma_{ap}N_{0}-(\sigma_{ap}+\sigma_{ep})N_{2}\right\}P_{p}^{\pm}$$

$$\begin{matrix} \frac{\partial N_{2}}{\partial z}=-\frac{N_{2}}{\tau}+\frac{\Gamma_{p}\lambda_{p}}{hcA_{co}}\left\{\sigma_{ap}N_{0}-(\sigma_{ap}+\sigma_{ep})N_{2}\right\}(P_{p}^{+}+P_{p}^{-}) \\ \text{ }+\frac{1}{hcA_{co}}\sum_{i=s,B} \Gamma_{i}\lambda_{i}\left\{\sigma_{ai}N_{0}-(\sigma_{ai}+\sigma_{ei})N_{2}\right\}P_{i} \end{matrix}$$

where ± indicates the positive and negative transmission directions of the pump light, respectively. *σ_ai_* and *σ_ei_*（*i*=*p、s、B*）represent the absorption cross-section and emission cross-section of pump light, signal light, and Stokes light respectively. *Г_i_*（*i*=*p、s、B*）is the effective overlap factor of pump, signal, and Stokes light with the doping area of the fiber core. *P_i_*（*i*=*p、s、B*）is the pump light, signal light, or Stokes light power, *h* is the Planck's constant, *τ* is the photon lifetime of the stimulated ytterbium ions and *A_co_* is the core area of the fiber.α_s_ and α*_B_* are the loss coefficient of signal light and Stokes light. *N*_0_ is the Yb-doped ion concentration. *f* is the SBS effect-induced noise, and its specific mathematical expression is shared in ref ^3,4^. κ_1_ and κ_2_ are the acoustic-optic field coupling coefficients of the SBS effect, which could be described by the following formula:

$$\begin{matrix} \kappa_{1}=\frac{\omega_{s}\gamma_{e}}{2n_{s}c\rho_{0}}, & \kappa_{2}=\frac{\omega_{s}\gamma_{e}}{2c^{2}\nu_{A}A_{eff}} \end{matrix}$$

*A_eff_* is the effective mode field area, which can be calculated according to the specific refractive index distribution.

Taking into account the impact of thermal loading on the Brillouin gain coefficient, we have adopted the approach from Ref [5] in our theoretical model to refine the Brillouin gain coefficient^5^. The temperature distribution *T*(z) along the longitudinal direction of the fiber is first calculated by the quantum loss and the heat conduction equation inside the fiber:

$$T(z)=\frac{\alpha_{p}(\lambda_{s}-\lambda_{p})\Delta P_{p}(z)}{4\pi k\lambda_{s}}(2\ln\frac{r_{clad}}{r_{core}}+\frac{2k}{Hr_{clad}})$$

*∆P_p_*(z) is the pump power for energy conversion, *k* is the thermal conductivities and *H* is the heat transfer coefficient. Then the Brillouin gain coefficient (*g*_B_) of the fiber is revised as a temperature-dependent function：

$$g_{T}(v,z)=\frac{g_{B}(\Delta v_{B}/2)^{2}}{((v+\kappa_{g}T(z))-v_{B}(z))^{2}+(\Delta v_{B}/2)^{2}}$$

∆*v*_B_ is the Brillouin gain bandwidth, *v*_B_ is the Brillouin frequency, and *κ*_g_ is the temperature coefficient.

Then the evolution of the Brillouin-Stokes light and signal light of the system can be obtained by applying the 4th-order Lunger Kuta method to solve the nonlinear amplitude coupled equations mentioned in this model. Defining the SBS threshold power as the output power when the return power reaches 1‰ of the output power, then the SBS threshold power of different fibers could be obtained.

1. *Theoretical models for TMI threshold power calculation*

We use the semi-analytical heat conduction equation to derive the mode instability evolution process in the fiber amplifier. Since the mode coupling in the fiber mainly occurs during the two modes *LP_01_* and *LP_11_*, only the two modes *LP_01_* and *LP_11_* in the fiber core are considered in the simulation model. Under the premise of considering only the *LP_01_* and *LP_11_* modes, the optical field in the fiber core can be expressed as:

where *E* represents the total optical field in the fiber core, and *a_0_* and *a_1_* are the mode amplitudes of the *LP_01_* and *LP_11_* modes. *β_0_*、*β_1_* are the transmission constants of these two modes. *ω_0_* and *ω_1_* are the angular frequencies of these two modes *ψ*_0_ and *ψ*_1_ represent the normalized optical field distributions of these two modes. Accordingly, the distribution of the light intensity in the fiber core could be described as:

$$I_{mn}=\frac{n_{0}}{2Z_{0}}a_{m}a_{n}^{*}$$

In the above equation, *Z*_0_ ≈ 377 Ω is the vacuum wave impedance. *I_0_* is the slow change term of the light intensity with respect to time, while is the interference term of the light intensity, i.e., the fast change term with respect to time. *β* and *Ω* represent the difference in the transmission constants and the difference in the angular frequencies of the *LP_01_* and *LP_11_* modes, respectively, which can be expressed as follows:

$$\beta=\beta_{0}-\beta_{1}\text{ ， }\Omega=\omega_{0}-\omega_{1}$$

Neglecting photon darkening and signal light background loss, the heat source in the gain fiber originates entirely from the quantum loss term of ytterbium ions. Assuming that the gain distribution in the doped fiber is *g*(*r*, *φ*, *z*, *t*), the heat source in the core of the gain fiber can be expressed in the following form:

Then considering the effect of the thermal loading and the fiber coiling on the refractive index distribution is considered. The thermal-induced refractive index change of a fiber depends on the temperature distribution. The cross-sectional temperature distribution of a YDF could be estimated through the analytical formula under the assumption of uniform heat distribution in fiber core^6^. Then, the thermal induced refractive index change in the fiber core and cladding could be expressed respectively as:

$$\Delta n_{co}\left(r\right)=\varepsilon\left[T_{0}-\frac{Qr^{2}}{4\pi a^{2}\kappa}\right]$$

$$\Delta n_{cl}\left(r\right)=\varepsilon\left[T_{0}-\frac{Q}{4\pi\kappa}-\frac{Q}{2\pi\kappa}In\left(\frac{r}{a}\right)\right]$$

where subscripts *co* and *cl* refer to the fiber core and cladding respectively, *ε* is the thermal optic coefficient, *T*_0_ is the central temperature, *Q* is the heat load, *a* is the core radius, and *κ* is the thermal conductivity.

A coiled fiber could be treated as a straight fiber and equivalent refractive index profile of the straight fiber could be expressed approximately as^7^:

$$n_{e}\left(x,y\right)=n_{0}\left(x,y\right)\left(1+\frac{x}{R_{f}R}\right)$$

where *n*_0_(*x*, *y*) and *n_e_*(*x*, *y*) are the original and equivalent refractive index profile of a coiled fiber respectively, *x* is the dimension in the coiling direction, *R_f_* is the equivalent coiling factor, and *R* is coiling radius of fiber.

Finally, the modified refractive index profile *n_m_* of a YDF under coiling and thermal effect could be expressed approximately as:

$$n_{m,co}=\left[n_{0,co}+\varepsilon\left(T_{0}-\frac{Qr^{2}}{4\pi a^{2}\kappa}\right)\right]\left(1+\frac{r\cdot\cos\theta}{R_{f}R}\right)$$

$$n_{m,cl}=\left[n_{0,cl}+\varepsilon\left(T_{0}-\frac{Q}{4\pi\kappa}-\frac{Q}{2\pi\kappa}In\left(\frac{r}{a}\right)\right)\right]\left(1+\frac{r\cdot\cos\theta}{R_{f}R}\right)$$

Bring Equations (6), (18), and (19) into the fluctuation equation:

The steady-state mode coupling equation for thermally induced mode instability can be expressed as:

where *χ*(Ω, *z*) is the nonlinear coupling coefficient between the fundamental and higher-order modes which can be expressed as:

By using the above model, the coupling evolution of *LP_01_* and *LP_11_* modes of the system can be obtained. Defining the TMI threshold power as the total output power when the maximum power share of *LP*_11_ mode is greater than 5% throughout the simulation calculation iterations, the TMI threshold power of different fibers can be obtained. The values of the main parameters used in the simulation are demonstrated in the following Table S1.

**Table S1. Main parameters (MP):**

| MP | Physical meaning | Value | MP | Physical meaning | Value |
| --- | --- | --- | --- | --- | --- |
| *NA* | Numerical aperture | 0.065 | *n_0_* | Cladding refractive index | 1.45 |
| *λ_s_* | Signal wavelength | 1064 nm | *λ_p_* | Pump wavelength | 976 nm |
| *σ_ap_* | Absorption cross-section of the pump laser | 1.77×10^-24^ m^2^ | *σ_ep_* | Emission cross-section of the pump laser | 1.71×10^-24^ m^2^ |
| *σ_as_* | Absorption cross-section of the signal laser | 6.38×10^-27^ m^2^ | *σ_es_* | Emission cross-section of the signal laser | 3.98×10^-25^ m^2^ |
| *σ_aB_* | Absorption cross-section of the Stokes light | 6.38×10^-27^ m^2^ | *σ_eB_* | Emission cross section of the Stokes light | 3.98×10^-25^ m^2^ |
| *Г_B_* | Acoustic damping rate | 2.056×10^8^s^-1^ | *ν_B_* | Frequency shift of the Stokes light | 16 GHz |
| *γ_e_* | Quartz electrostrictive constant | 0.902 | *ρ_0_* | Quartz density | 2210 kg·m^-3^ |
| *p_s_* | Signal power | 10 | *v_A_* | Sound velocity | 5893.1 m·s^-1^ |
| *lc* | Bending length | 0.9×*L_YDF_* | *r_c_* | Bending radius | 0.3 m |
| *κ* | Thermal conductivity | 1.38 W·(Km)^-1^ | *k_g_* | Temperature coefficient | 8×10^5^ Hz·K^-1^ |
| *R_f_* | Equivalent coiling factor | - | *ε* | Thermal optic coefficient | 1.2×10^-5^ K^-1^ |
| τ | Lifetime of stimulated Yb ion | 901 µs |  |  |  |

**Section III**

In this section, we analyzed the effects of key parameter variations (Δ*L*_1_, Δ*L*_3_, Δ*n*_1_, Δ*n*_2,_ and Δ*n*_3_) the effective mode area of the fundamental mode, and the bending loss coefficient of *LP*_11_ mode by using the finite element simulation. This analysis provides a clear understanding of the parameters design tolerances and fabrication challenges of the bat-type refractive index distribution. Based on the simulation results, we proposed a feasible fiber parameter design scheme for realizing a kilowatt-class single-frequency fiber laser, as summarized in Table 2 of the manuscript. The following are the specific parameters analysis and selection process:

For further improving the performance of the active fiber in simultaneously suppressing the SBS and TMI effects under high thermal load, a bat-type refractive index distribution was applied as shared in figure S2. According to the theoretical study, focusing the Yb-doped core diameter at 35 μm is an ideal design to balance the suppression of SBS and TMI effects, so the Δ*L*_2_ is fixed at 35 μm in the finite element simulation. Besides, to keep the average core NA near 0.03, the maximum refractive index of the center region is designed at 1.4505. In the finite element simulation model, we have investigated the effect of other main parameters (Δ*L*_1_, Δ*L*_3_, Δ*n*_1_, Δ*n*_2,_ and Δ*n*_3_) of the bat-type refractive index distribution on the characteristics of the active fiber including the effective mode field area of the fundamental mode and the higher-order modes loss coefficients.


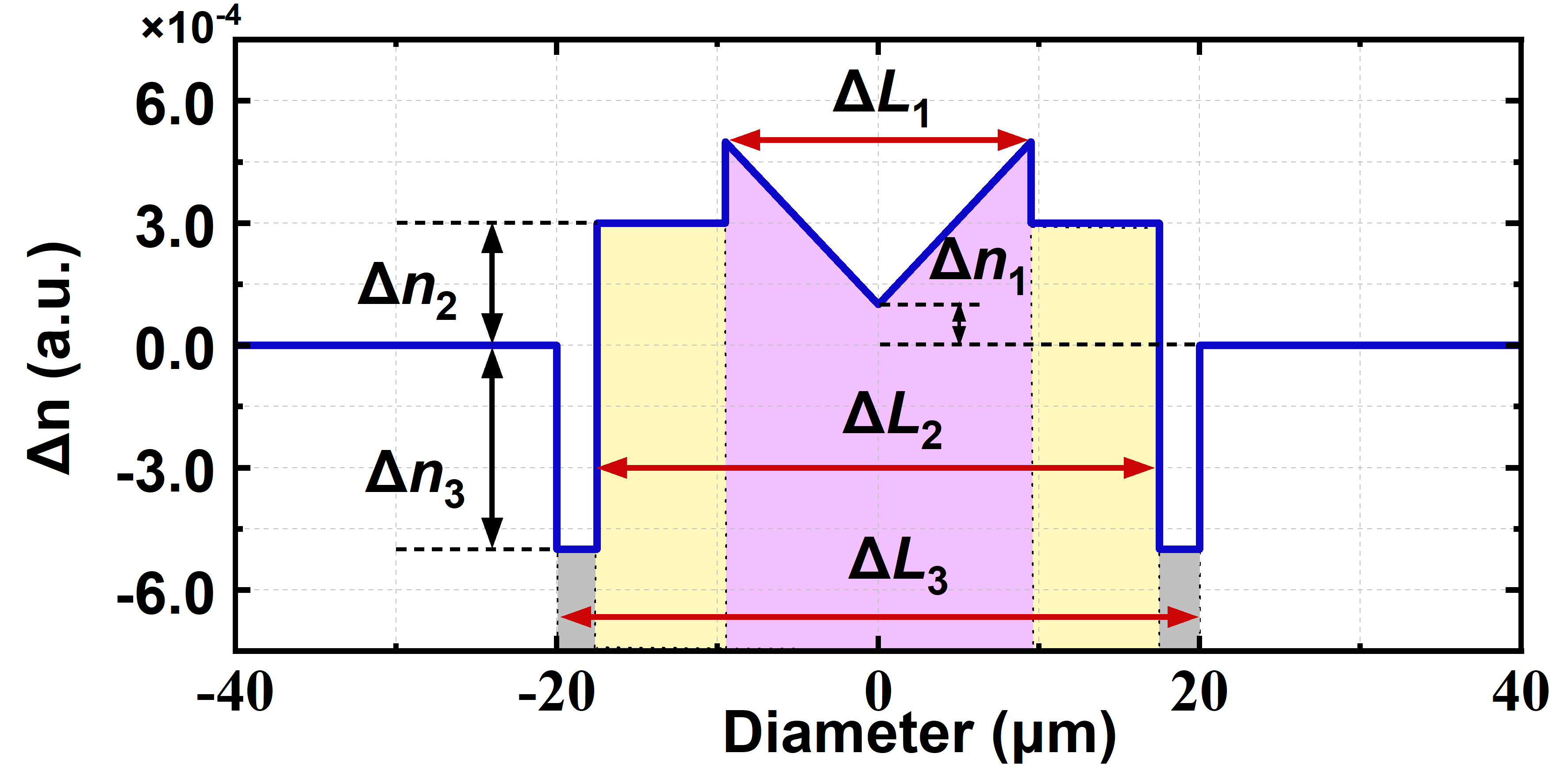


**Fig. S2** The refractive index distribution of ultralow *NA* active fibers.

Based on the aforementioned theoretical models, it is demonstrated that achieving a stimulated Brillouin scattering (SBS) threshold exceeding 1000 W requires the mode field area of the *LP*_01_ mode to be greater than 651.0 μm² while attaining a transverse mode instability (TMI) threshold above 1000 W necessitates an average bending loss coefficient of the *LP*_11_ mode exceeding 27.5 dB·m^-1^. To address this requirement, the average thermal power intensity was set to 40.1 W·m^-1^ in the finite element simulation, corresponding to the theoretical average heat load for a kilowatt-level fiber amplifier utilizing this bat-type ytterbium-doped fiber (YDF), as demonstrated in fig. S3. Then the bending radius was set to 0.25 m. Building on this configuration, the theoretical tolerance ranges for the fiber design parameters (Δ*L*_1_, Δ*L*_3_, Δ*n*_1_, Δ*n*_2,_ and Δ*n*_3_) were also discussed, as detailed in the following sections.


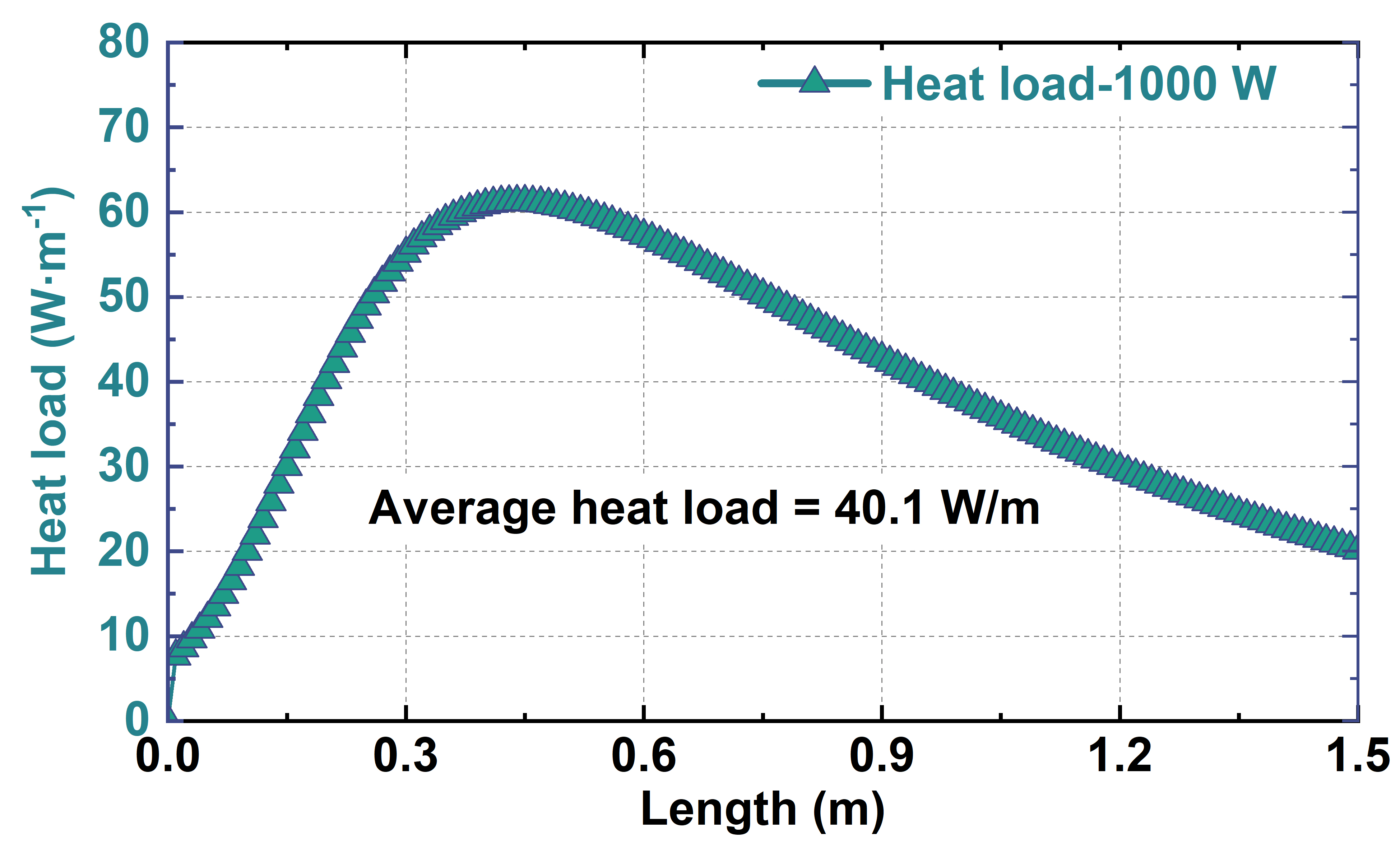


**Fig. S3** Heat load of the bat-type YDF with an output power of 1000 W.

Fig. S4 illustrates the effective mode area of the fundamental mode and the bending loss coefficient of the *LP*_11_ mode when Δ*L*_1_ is different. As shown in Fig. S4, the effective mode area of the fundamental mode increases, while the bending loss coefficient of the *LP*_11_ mode first increases and then decreases when Δ*L*_1_ increases from 16.0 μm to 24.0 μm. The effective mode area of the fundamental mode could exceed 651.0 μm^2^ when Δ*L*_1_ ranges from 16.4 μm to 24.0 μm, and the bending loss coefficient of the *LP*_11_ mode could exceed 27.5 dB·m^-1^ when Δ*L*_1_ ranges from 16.0 μm to 20.8 μm. **Accordingly, the design requirement of Δ*L*_1_ is 18.6 ± 2.2 μm.**


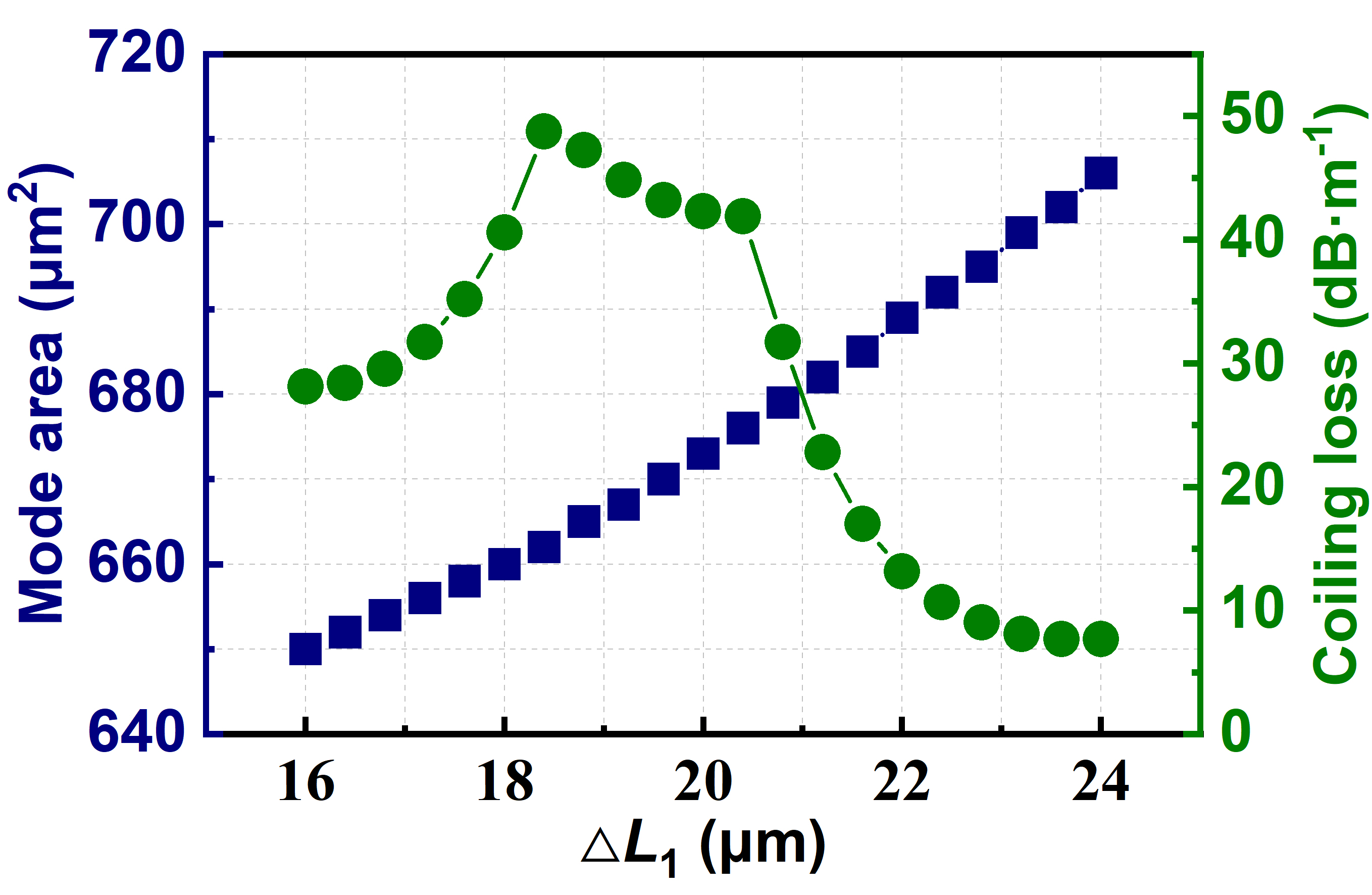


**Fig. S4** Effective mode area of the fundamental mode and bending loss coefficient of the *LP*_11_ mode when Δ*L*_1_ is different.

Fig. S5 illustrates the effective mode area of the fundamental mode and the bending loss coefficient of the *LP*_11_ mode when Δ*L*_3_ is different. As shown in Fig. S5, the effective mode area of the fundamental mode decreases with the increases of Δ*L*_3_. The bending loss coefficient of the *LP*_11_ mode first increases then keeps 45 ~dB·m^-1^, and finally decreases when Δ*L*_3_ increases from 36 μm to 44 μm. The effective mode area of the fundamental mode could exceed 651.0 μm^2^ when Δ*L*_3_ ranges from 36.0 μm to 41.6 μm, and the bending loss coefficient of the *LP*_11_ mode could exceed 27.5 dB·m^-1^ when Δ*L*_3_ ranges from 38.4 μm to 41.2 μm. **Accordingly, the design requirement of Δ*L*_3_ is 39.8 ± 1.4 μm.**


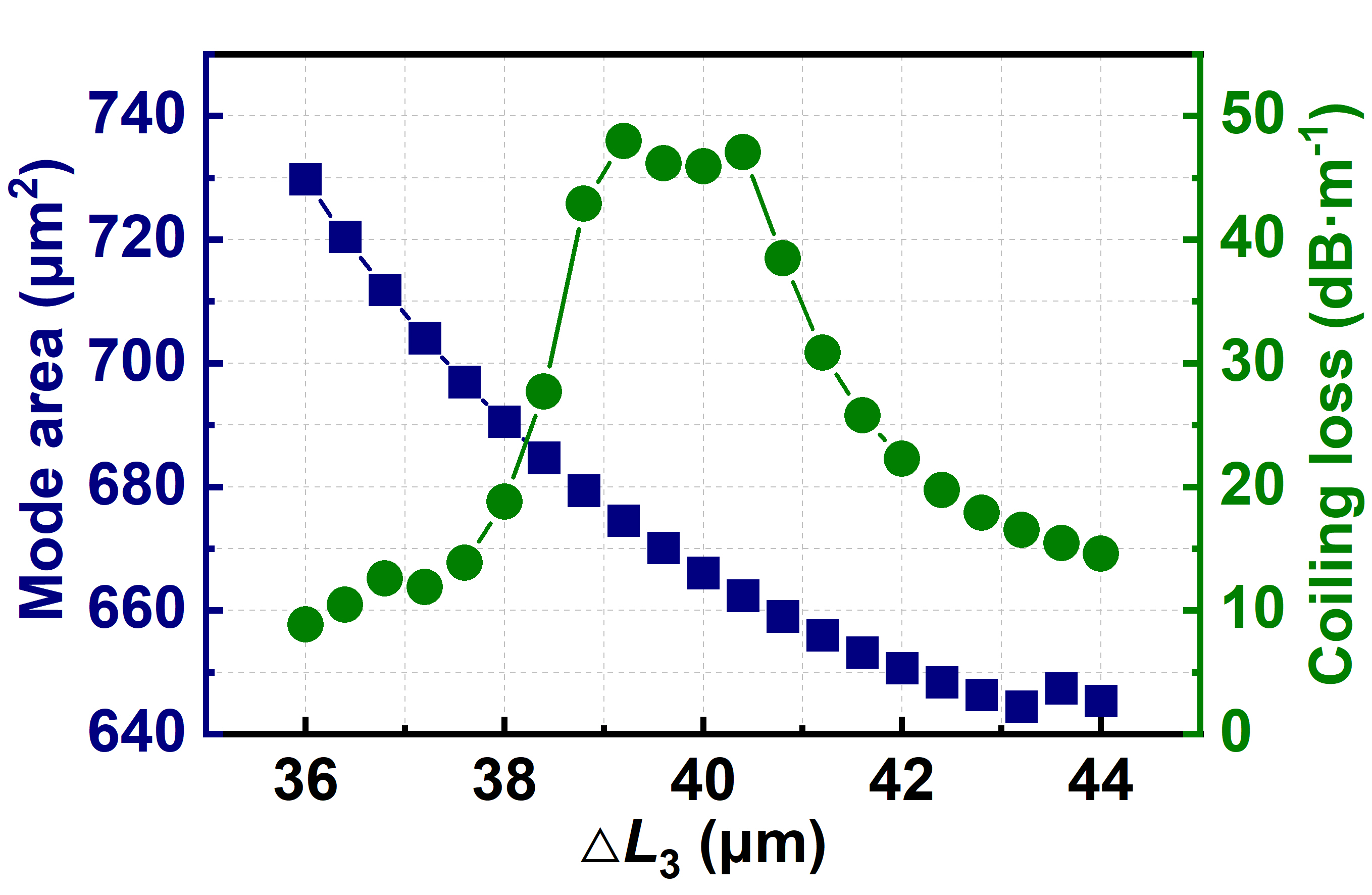


**Fig. S5** Effective mode area of the fundamental mode and bending loss coefficient of the *LP*_11_ mode when Δ*L*_3_ is different.

Fig. S6 illustrates the effective mode area of the fundamental mode and the bending loss coefficient of the *LP*_11_ mode when Δ*n*_1_ is different. As shown in Fig. S6, the effective mode area of the fundamental mode decreases, while the bending loss coefficient of the *LP*_11_ mode first increases, then decreases when Δ*n*_1_ increases from 0 to 3.0×10^-4^. The effective mode area of the fundamental mode could exceed 651.0 μm^2^ when Δ*n*_1_ ranges from 0 to 1.3×10^-4^, and the bending loss coefficient of the *LP*_11_ mode could exceed 27.5 dB·m^-1^ when Δ*n*_1_ ranges from 0 to 2.2×10^-4^. **Accordingly, the design requirement of Δ*n*_1_ is 1.1± 1.1×10^-4^.**


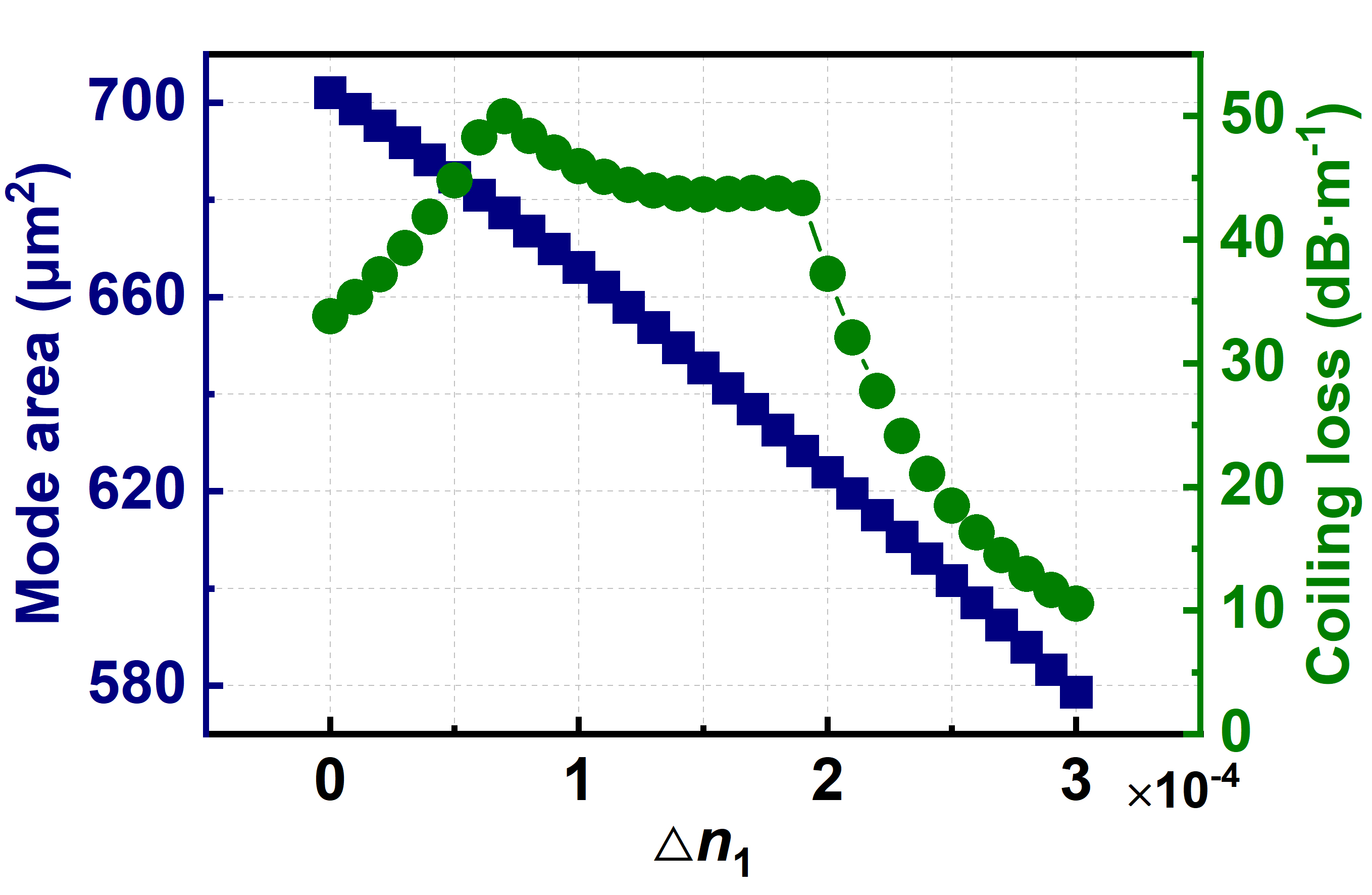


**Fig. S6** Effective mode area of the fundamental mode and bending loss coefficient of the *LP*_11_ mode when Δ*n*_1_ is different.

Fig. S7 illustrates the effective mode area of the fundamental mode and the bending loss coefficient of the *LP*_11_ mode when Δ*n*_2_ is different. As shown in Fig. S7, the effective mode area of the fundamental mode increases, while the bending loss coefficient of the *LP*_11_ mode shows a fluctuating decrease when Δ*n*_2_ increases from 2.0×10^-4^ to 4.0×10^-4^. The effective mode area of the fundamental mode could exceed 651.0 μm^2^ when Δ*n*_2_ ranges from 2.8×10^-4^ to 4.0×10^-4^, and the bending loss coefficient of the *LP*_11_ mode could exceed 27.5 dB·m^-1^ when Δ*n*_2_ ranges from 2.0×10^-4^ to 3.1×10^-4^. **Accordingly, the design requirement of Δ*n*_2_ is 2.95 ± 0.15×10^-4^.**


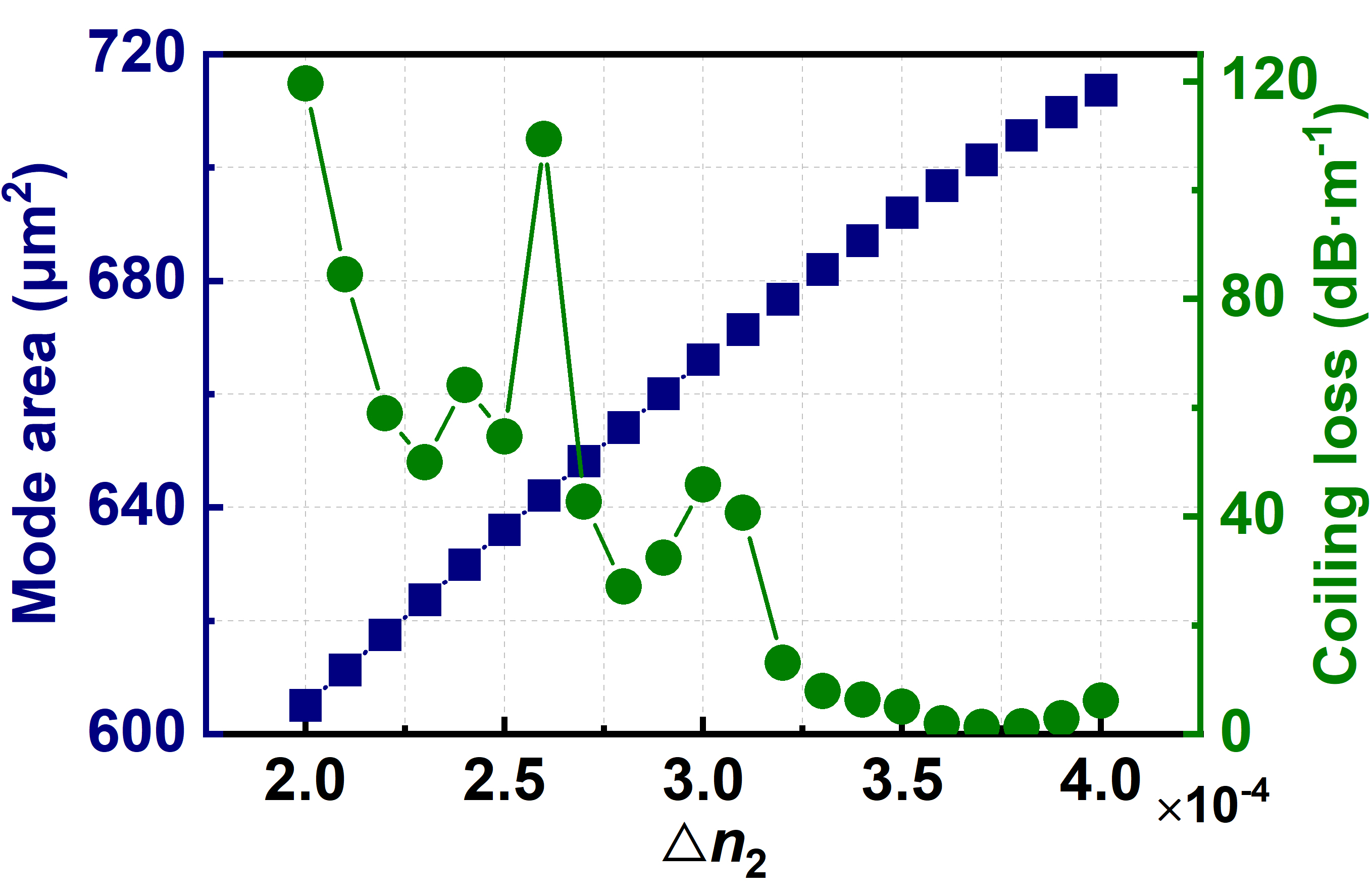


**Fig. S7** Effective mode area of the fundamental mode and bending loss coefficient of the *LP*_11_ mode when Δ*n*_2_ is different.

Fig. S8 illustrates the effective mode area of the fundamental mode and the bending loss coefficient of the *LP*_11_ mode when Δ*n*_3_ is different. As shown in Fig. S8, the effective mode area of the fundamental mode decreases with the increases of Δ*n*_3_. The bending loss coefficient of the *LP*_11_ mode first increases then keeps ~45 dB·m^-1^, and finally decreases when Δ*n*_3_ increases from 0 to -7.0×10^-4^. The effective mode area of the fundamental mode could exceed 651.0 μm^2^ when Δ*n*_3_ ranges from 0 to -6.2×10^-4^, and the bending loss coefficient of the *LP*_11_ mode could exceed 27.5 dB·m^-1^ when Δ*n*_3_ ranges from -3.8×10^-4^ to -6.0×10^-4^. **Accordingly, the design requirement of Δ*n*_3_ is -4.9 ± 1.1×10^-4^.**


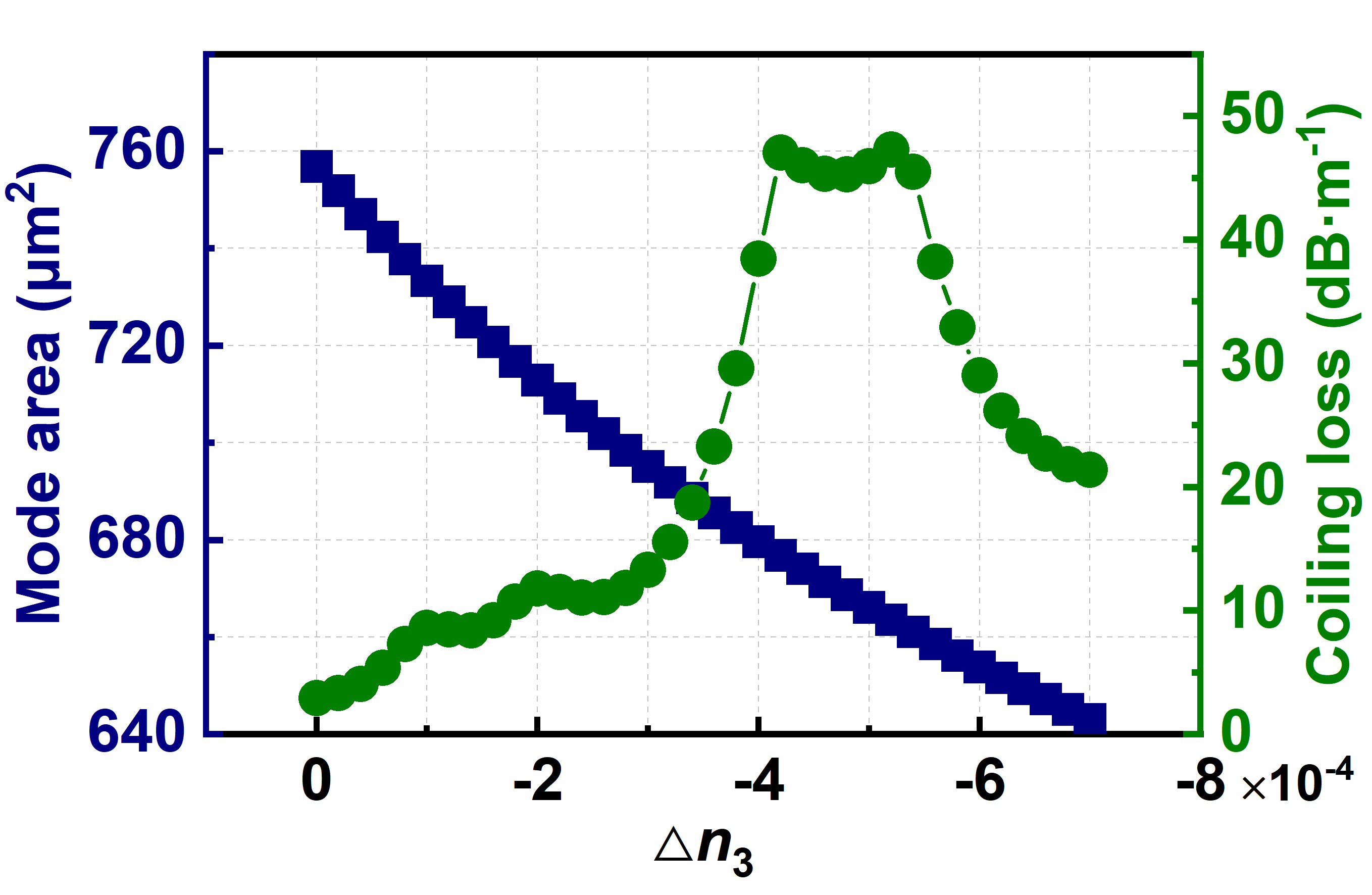


**Fig. S8** Effective mode area of the fundamental mode and bending loss coefficient of the *LP*_11_ mode when Δ*n*_3_ is different.

In conclusion, the simulation results reveal that Δ*n*_2_ has the most stringent tolerance requirement, with a permissible refractive index deviation of only ±0.15×10⁻⁴. This parameter requires precise control over ion deposition rate, dopant concentration, and temperature stability during the fiber fabrication process. Despite our efforts to achieve precise control over Δ*n*_2_, practical challenges arise during the preform collapse process, where uncontrollable ion diffusion can lead to deviations from the target refractive index profile. As shown in Fig. 3c of the manuscript, although the average refractive index of the stepped region is controlled within the required range, the fabricated fiber exhibits a less flat refractive index profile in this region compared to the design, which leads to the decrease of bending loss coefficient of the *LP*_11_ mode (design: 47.5 dB·m^-1^; reality: 36.4 dB·m^-1^). This discrepancy underscores the difficulty of maintaining the ideal refractive index distribution in practice.

**Section IV**

In this section, we have performed detailed simulations based on the actual measured refractive index profile of the fiber. These simulations analyze the thermal load, bending loss characteristics, and the power evolution of the *LP*_01_ and *LP*_11_ modes during the power amplification. For comparison, we have also included a conventional SIF with an NA of 0.032 as a control group.

The theoretical simulation process can be summarized as follows:

1. **Finite Element Simulation**: We simulated the mode field distribution under different thermal loads and calculated the corresponding changes in the mode field area and bending loss, the results are demonstrated in the following figure. The simulation results show that with an increase in heat load, the mode field area of both fibers performs a slight decrease. Meanwhile, the bending loss coefficient of *LP*_11_ mode for both fibers demonstrates a fluctuating decrease with the increase of heat load. However, it is worth noting that the bat-type refractive index fibers have a significantly higher bending loss coefficient under the heat load below 80 W·m^-1^.


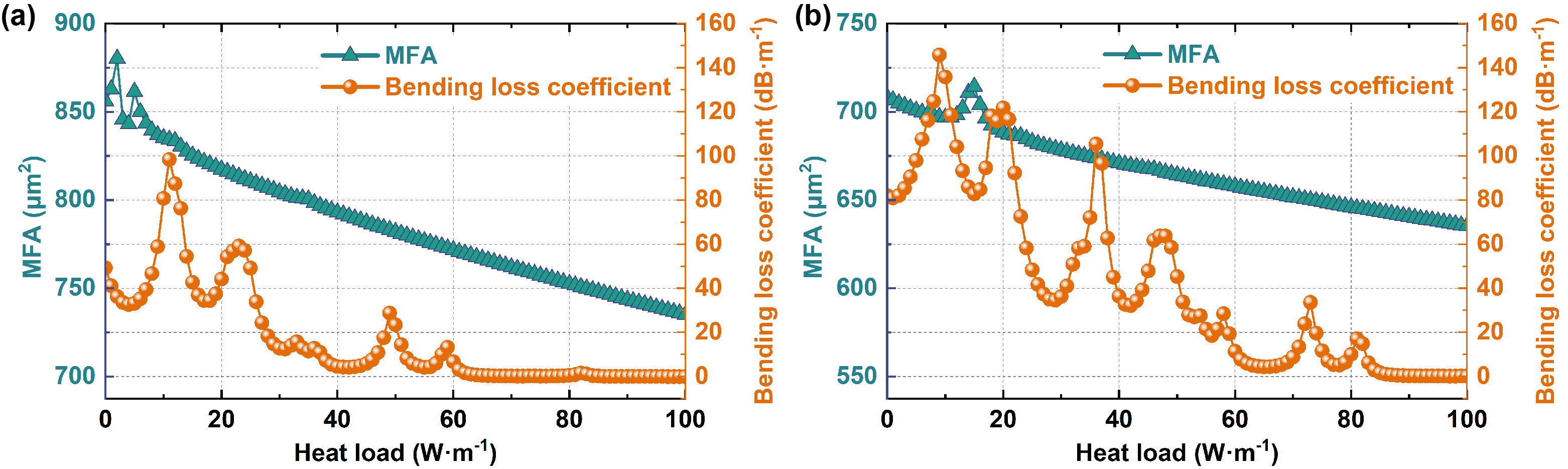


**Fig. S9** The mode field area (MFA, *LP*_01_ mode) and the bending loss coefficient (*LP*_11_ mode) versus the heat load: (a) in the conventional SIF; (b) in the bat-type refractive index fiber.

1. **Thermal Load and Bending Loss Coefficient Analysis**: We calculated the actual heat load distribution along the fiber under different pump powers and determined the bending loss distribution of the *LP*_11_ mode along the fiber. The following figure demonstrates the calculating results when the amplifier is operating around the TMI threshold (770.2 W for the SIF; 1103.7 W for the HOMLF). The simulation results demonstrate that the bat-type refractive index fibers have a significantly higher bending loss coefficient even under a higher heat load.


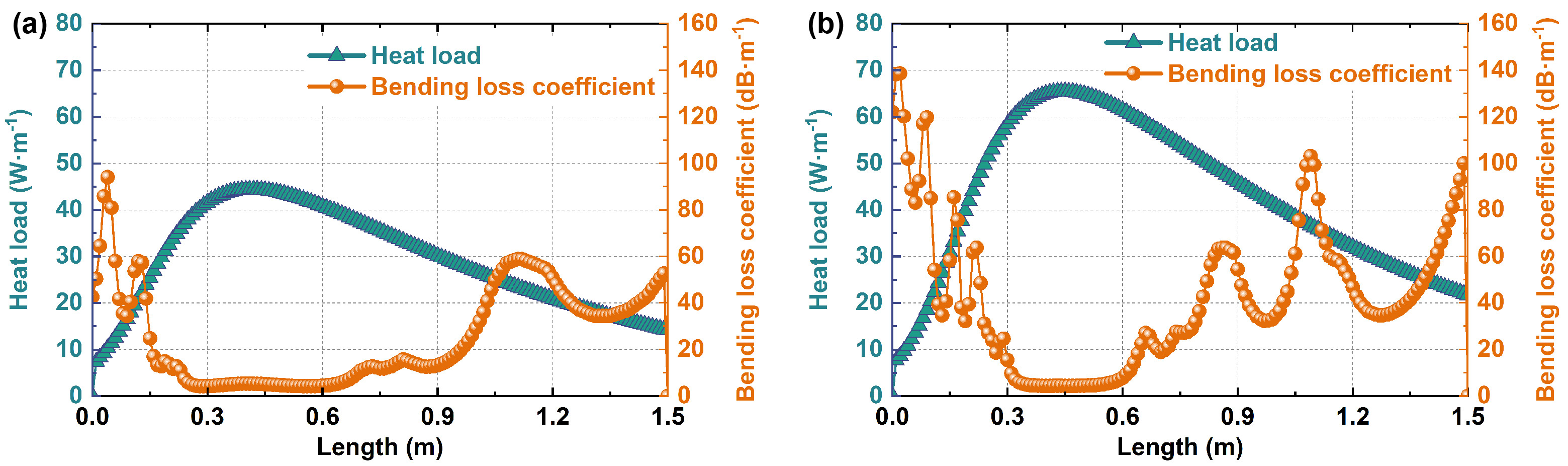


**Fig. S10** The mode field area (MFA, *LP*_01_ mode) and the bending loss coefficient (*LP*_11_ mode) distribution in (a) the conventional SIF or (b) the bat-type refractive index fiber.

1. **Modes Coupling simulation**: By incorporating the bending loss distribution into the mode coupling model, the energy distribution of the *LP*_01_ and *LP*_11_ modes along the fiber could be iteratively calculated. As the following figure shows, benefiting from the better mode loss characteristics of the bat-type refractive index fibers under thermal loading, it performs better in suppressing the energy transfer from *LP*_01_ mode to *LP*_11_ mode.


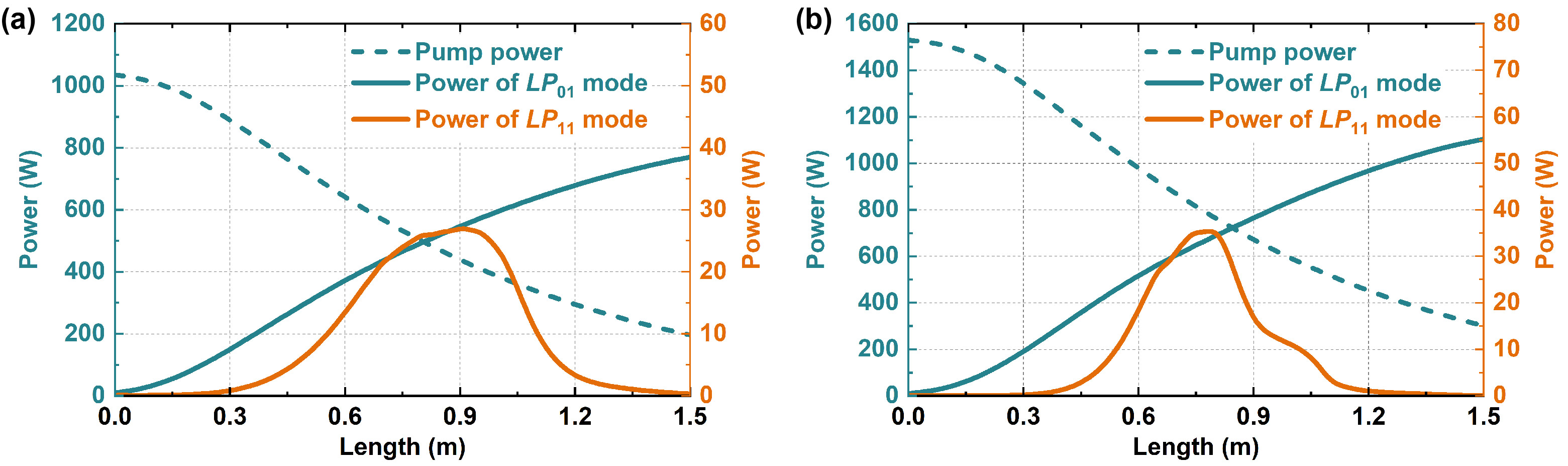


**Fig. S11** The power curve of the *LP*_01_ mode and *LP*_11_ mode: (a) conventional SIFs; (b) bat-type refractive index fibers.

In conclusion, the bat-type refractive index profile could achieve a higher bending loss coefficient for the *LP*_11_ mode under high heat load, which significantly suppresses the amplification of HOMs and contributes to a higher TMI threshold. However, it is important to note that if the amplifier operates beyond the TMI threshold, strong mode coupling can convert significant energy of the *LP*_01_ mode into the *LP*_11_ mode. And due to the high bending loss coefficient in the bat-type refractive index fibers, these HOMs will be effectively filtered out, leading to a reduction in power conversion efficiency and possible output power stagnation.

Section V:

In this section, we elaborated on the methodology for linewidth estimation and present the evolution of linewidth during the power amplification process.

The direct detection of laser linewidth by using the delayed self-heterodyne principle is traditionally carried out by utilizing a Mach-Zehnder interferometer structure shown in Fig.S12^8,9^. The injected single-frequency laser is split into two beams by the 1 × 2 coupler 1. One beam is delayed by the single-mode fiber. The other beam goes through the acoustic optical modulator (AOM) to generate a frequency shift. The two beams are recombined by the coupler 2 and their beating signal is detected by the photodetector (PD). The detected voltage signal is then transported to the electric spectrum analyzer (ESA). The output power spectrum of the delayed self-heterodyne interferometer (DSHI) can be given by:

$$S\left(f,\Delta f\right)=S_{1}\cdot S_{2}+S_{3}$$

here

$$S_{2}=1-\exp(-2\pi\cdot\Delta f\cdot\tau_{d})\cdot\left[\cos\left[2\pi(f\pm f_{1})\tau_{d}\right]+\Delta f\cdot\frac{\sin\left[2\pi(f\pm f_{1})\tau_{d}\right]}{f\pm f}\right]$$

$$S_{3}=\frac{\pi E_{0}^{2}}{2}\cdot\exp(-2\pi\cdot\Delta f\cdot\tau_{d})\cdot\delta(f\pm f_{1})$$

where *E*_0_ is the amplitude of the two beams, *f* is the measurement frequency, *f*_1_ is the frequency shift provided by the AOM, *τ*_d_ (*τ*_d_ = *L*·*c*^-1^, *L* is the length of delayed fiber and *c* is the speed of light) is the time delay of one path with respect to the other path, and *∆f* is the fullwidth-half-maximum (FWHM) of the power spectrum. *S*_1_ is the Lorentzian spectrum of the injected laser and *S*_2_ is the periodic modulation power spectrum. For equation (5), when *f* ≠ *f*_1_, *δ(f* ± *f*_1_) = 0, *S*_3_ = 0, so that the power spectrum *S* can be simplified to be *S( f, ∆f )* = *S*_1_·*S*_2_.

When the delayed length is less than the laser coherent length, the output power spectrum *S* is the product of the Lorentzian spectrum *S*_1_ and the periodic modulation power spectrum *S*_2_. In the output power spectrum *S*, the shorter delayed length and narrower laser linewidth will lead to better contrast and a more obvious coherent envelope of the simulation power spectrum. According to the above shared theoretical analysis, the amplitude difference of the coherent envelope is a fixed value once the fiber delayed length and the laser linewidth are determined. Conversely, we can choose the value of contrast difference between peaks and troughs (CDPT) of the coherent envelope of the power spectrum at a certain frequency to reflect the laser linewidth. Since the periodicity of the coherent envelope is determined by the periodic modulation power spectrum *S*_2_, the model of CDPT can be established as:

$$\begin{matrix} \Delta S(\Delta f)=10\log_{10}S_{peak}-10\log_{10}S_{trough} \\ =10\log_{10}S(f_{1}-\frac{2l-1}{2}\frac{c}{nL},\Delta f)\cdot10\log_{10}S(f_{1}-\frac{mc}{nL},\Delta f) \end{matrix}$$

where *n* is the fiber optic refractive index and *c* is the speed of light. The parameters *l* = 2*,* 3*,* 4 *. . . . . .* and *m* = 1*,* 2*,* 3 *. . . . . .* represent position of different peaks and troughs, respectively. For instance, if we want to get the value of the second CDSP (*∆S*), the parameters *l* and *m* should be set to 2 and 2, respectively. On the contrary, the linewidth of the measured laser can also be estimated from the value of *∆S*.


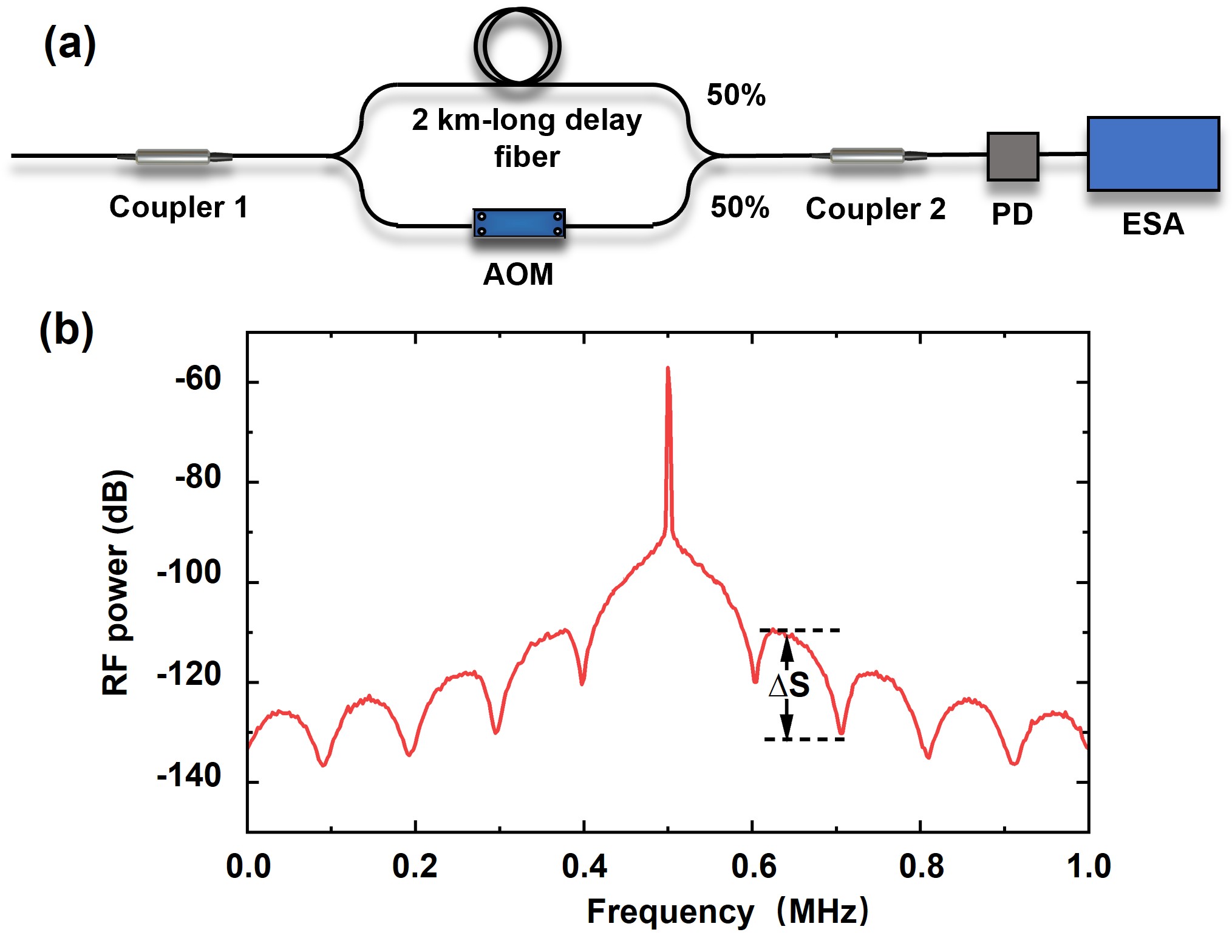


**Fig. S12** (a) Schematic of the delayed self-heterodyne interferometer to measure laser linewidth; (b) the output power spectrum of the DSHI

Besides, in our experiment, the broadening of the laser linewidth is primarily attributed to two factors. Firstly, during the amplification process, the noise introduced by the pump laser is coupled into the signal light through the amplitude-to-phase modulation (AM-to-PM) effect, leading to linewidth broadening^10,11^. In our experiments, attributed to the pump laser introduced in the cascaded pre-amplifiers and the main amplifier, the laser linewidth increased to ~1.0 kHz.

The second factor contributing to the linewidth broadening is the stimulated Brillouin scattering (SBS) effect in the main amplifier. As the SBS effect introduces additional noise and phase fluctuations, we observed a significant broadening of the signal laser linewidth. The extent of this broadening is directly related to the strength of the SBS effect, with stronger SBS leading to more pronounced linewidth broadening. The detailed linewidth information is shared in figure S13.


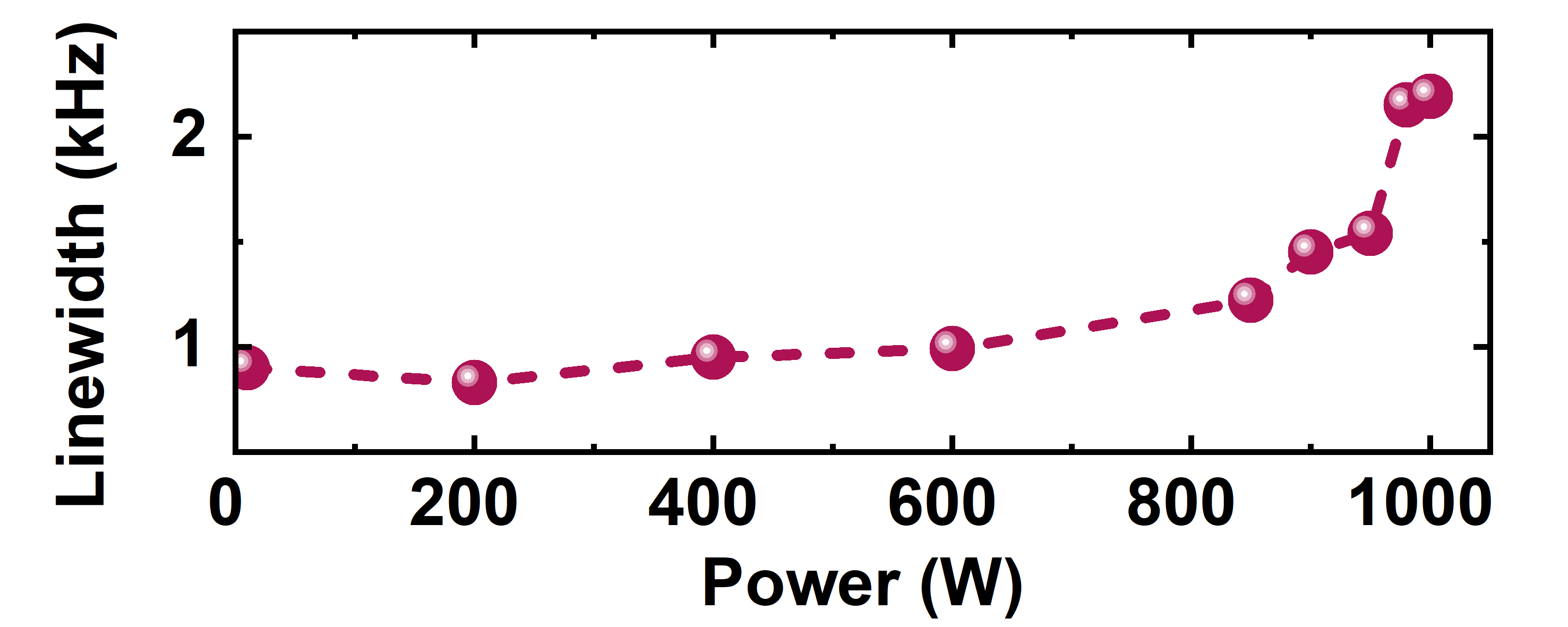


**Fig. S13** The linewidth of the output laser.

**Section VI**

In this section, we gave out detailed information about the output laser, including the long-term stability, polarization extinction ratio (PER), phase noise, and pointing noise.

1. Long-term stability

We have conducted a stability test on the laser system, with a continuous operation time exceeding one hour and the results are shared in Fig. S14. The test results demonstrate that the amplifier can operate stably with a power fluctuation of approximately 2.2% at the highest output power level.


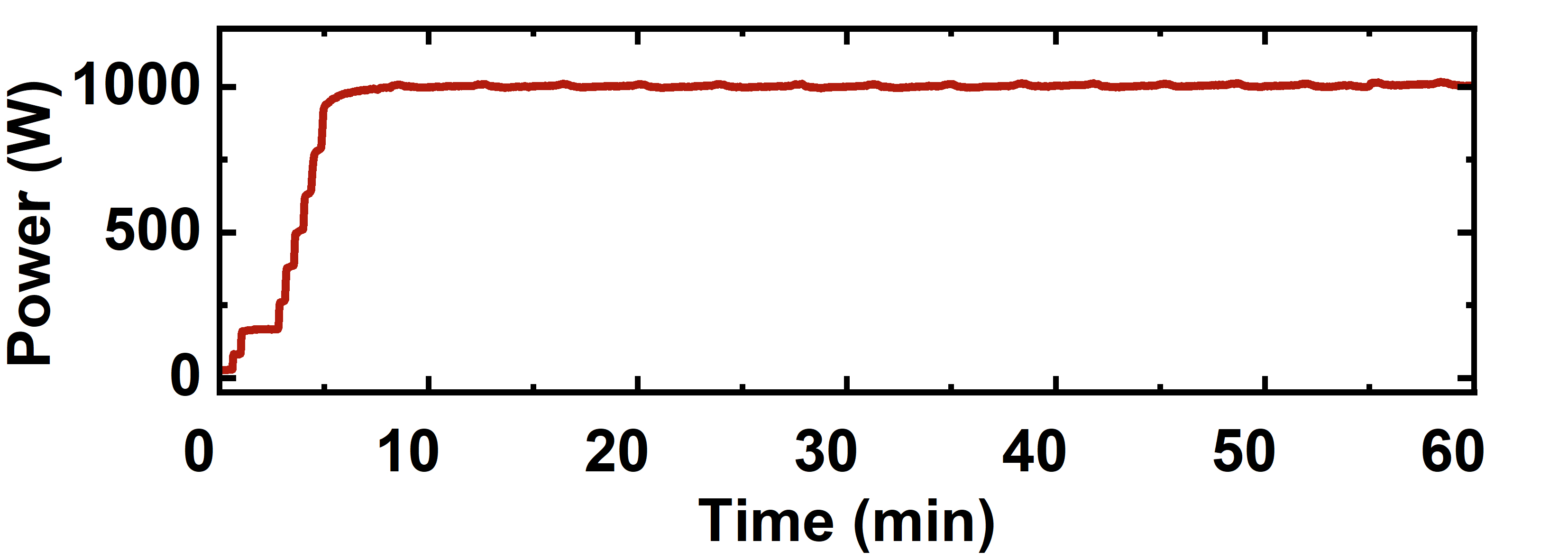


**Fig. S14** The power fluctuation of the output laser.

2. Polarization extinction ratio

Our single-frequency fiber laser system incorporates a polarization-maintaining (PM) seed laser and a PM pre-amplifier, ensuring that the laser injected into the main amplifier is polarization-maintained with a PER exceeding 20 dB. Due to the relatively short length of the active fiber used in the main amplifier, the polarization state of the signal laser can be maintained to a certain extent. During our experiments, we conducted a one-hour stability test on the output laser with an output power of kilowatt level, and the PER was observed to remain around 18 dB, as Fig. S15 shows.


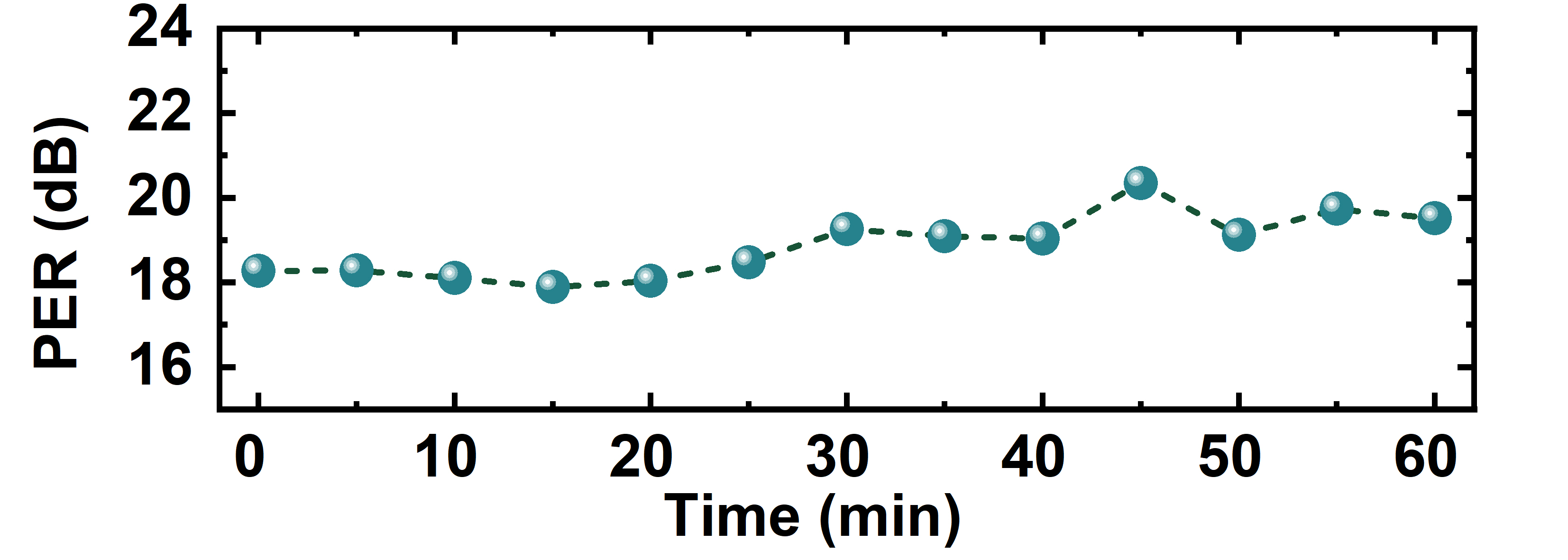


**Fig. S15** The polarization extinction ratio (PER) of the output laser.

3. Phase noise

Fig. S16 illustrates the phase noise of the seed laser, the cascade preamplifier, and the output laser. As can be seen from the figure, the phase noise of the output laser is well maintained at the frequency range of 1 Hz to 10 kHz throughout the amplification process, and no significant noise degradation is observed compared to the phase noise of the seed laser. However, it should be noted that the phase noise exhibits significant degradation in the frequency range above 100 kHz under high-power operation, which is attributed to the Stimulated Brillouin Scattering effect.


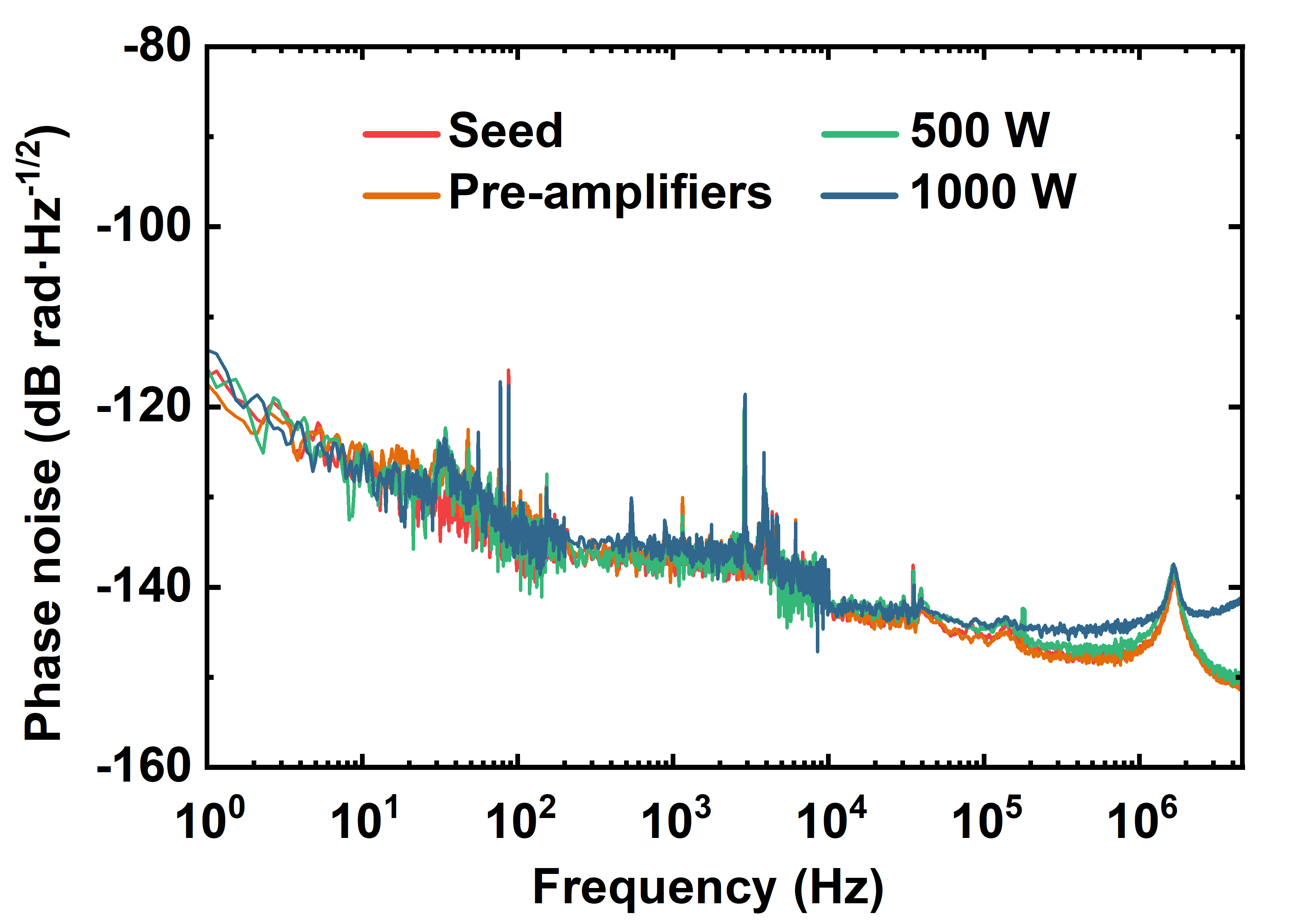


**Fig. S16** The phase noise of the output laser.

4. Pointing noise

The pointing noise of the output laser is measured based on a four-quadrant photodetector (Thorlabs PDQ80A in corporation with an electric driver Thorlabs KPA101). Fig.S17 illustrates the results of pointing noise for the x direction and y direction. As can be seen from the figure, the intensity of the pointing noise exhibits a fluctuating downward trend as the frequency increases and finally decreases to ~1.8×10^-5^ Hz^1/2^ at the frequency of 10 kHz for both x and y directions.


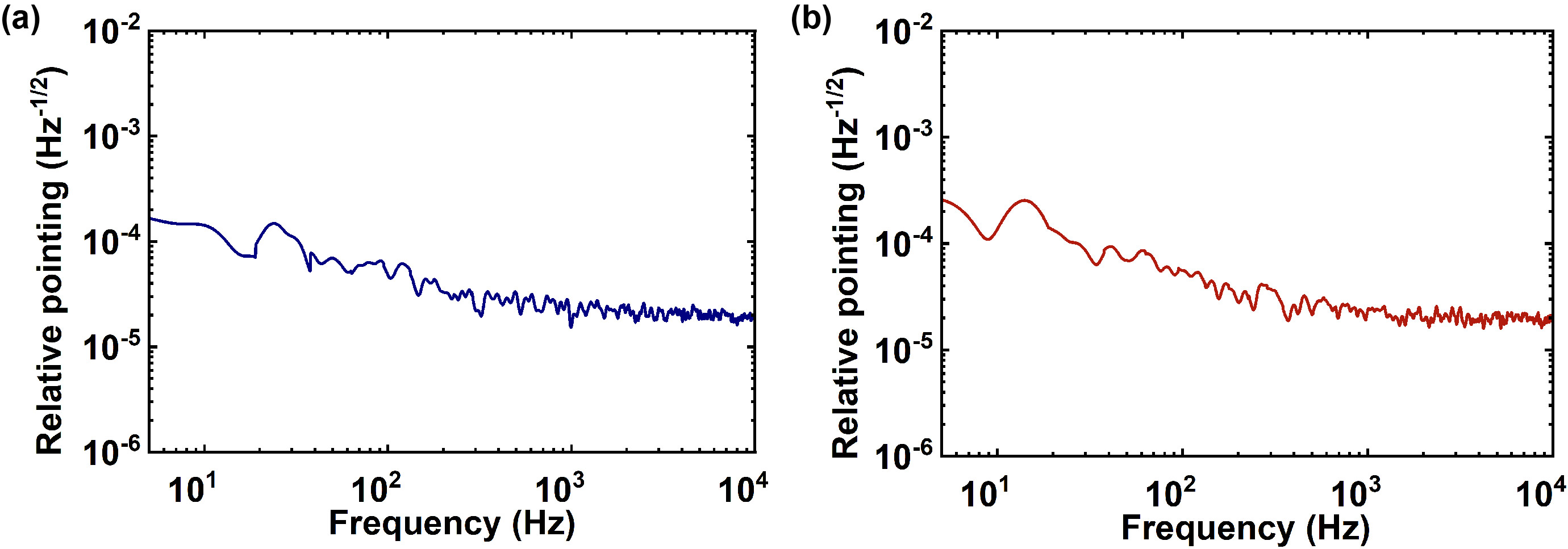


**Fig. S17** The pointing noise of the output laser. (a) x direction; (b) y direction.

**Reference**

1 Agrawal & Govind. *Nonlinear Fiber Optics (Fifth Edition)*. (Nonlinear Fiber Optics, 2013).

2 Tao, R. et al. Suppressing mode instabilities by optimizing the fiber coiling methods. *Laser Physics Letters* **14**, 025101 (2017).

3 Jenkins, R. B., Sova, R. M. & Joseph, R. I. Steady-State Noise Analysis of Spontaneous and Stimulated Brillouin Scattering in Optical Fibers. *Journal of Lightwave Technology* **25**, 763-770 (2007).

4 Boyd, R. W., Rzaewski, K. & Narum, P. Noise initiation of stimulated Brillouin scattering. *Physical Review A* **42**, 5514-5521 (1990).

5 Lou, Z. et al. Increasing the SBS threshold by applying a flexible temperature modulation technique with temperature measurement of the fiber core. *Optics Express* **28**, 13323-13335 (2020).

6 Brown, D. C. & Hoffman, H. J. Thermal, stress, and thermo-optic effects in high average power double-clad silica fiber lasers. *IEEE Journal of Quantum Electronics* **37**, 207-217 (2001).

7 Schermer, R. T. & Cole, J. H. Improved Bend Loss Formula Verified for Optical Fiber by Simulation and Experiment. *IEEE Journal of Quantum Electronics* **43**, 899-909 (2007).

8 Huang, S. *et al.* Laser Linewidth Measurement Based on Amplitude Difference Comparison of Coherent Envelope. *IEEE Photonics Technology Letters* **28**, 759-762 (2016).

9 Huang, S. *et al*. Precise measurement of ultra-narrow laser linewidths using the strong coherent envelope. *Scientific Reports* **7** (2017).

10 Zhang, Q. *et al.* Pump RIN coupling to frequency noise of a polarization-maintaining 2 microm single frequency fiber laser. *Optics Express* **29**, 3221-3229 (2021).

11 Li, C., Xu, S., Yang, C., Wei, X. & Yang, Z. Frequency noise of high-gain phosphate fiber single-frequency laser. *Laser Physics* **23**, 045107 (2013).
